# Benzotriazole in Cancer: A Systematic Review on Preclinical Evidence and Structure–Activity Relationship

**DOI:** 10.3390/ph19010077

**Published:** 2025-12-30

**Authors:** Gabriel Mardale, Alexandra Prodea, Andreea Munteanu, Mihaela Jorgovan, Sabina Mardale, Victor Cristian Dumitrascu, Codruța Șoica

**Affiliations:** 1 Faculty of Pharmacy, “Victor Babes” University of Medicine and Pharmacy, Eftimie Murgu Square, No. 2, 300041 Timișoara, Romania; mardale.gabriel@umft.ro (G.M.); andreea.milan@umft.ro (A.M.); mihaela.coban@umft.ro (M.J.); codrutasoica@umft.ro (C.Ș.); 2 Research Center for Experimental Pharmacology and Drug Design (X-Pharm Design), “Victor Babes” University of Medicine and Pharmacy, Eftimie Murgu Square, No. 2, 300041 Timișoara, Romania; 3 Timiș County Emergency Clinical Hospital Pius Brinzeu, Liviu Rebreanu 156, 300723 Timișoara, Romania; sabinabilavu@yahoo.com (S.M.);

**Keywords:** benzotriazole, anticancer, TBB, cytotoxicity, apoptosis

## Abstract

**Background:** A benzotriazole is a heterocycle frequently used in medicinal chemistry to obtain potent drug candidates, including anticancer agents. Nonetheless, the available literature lacks a comprehensive review of the in vitro and in vivo studies regarding these derivatives. Thus, our study aims to review the preclinical evidence on benzotriazole derivatives that showed potential as anticancer candidates, focusing on the cytotoxicity, mechanisms of action, structure–activity relationship, and methodological rigor of the included studies. **Methods:** We searched PubMed, Scopus, and Web of Science and included 41 studies in our analysis following the selection process. Additionally, we assessed the risk of bias using the QUIN tool for in vitro and the SYRCLE tool for in vivo studies in order to assess the methodological rigor of the included studies. **Results:** The benzotriazole derivatives were classified according to their structure in four classes, namely N-derivatives, C-derivatives, fused derivatives, and organometallic compounds. The in vitro results showed that certain derivatives, such as halogen, alkyl-aryl, or natural-base hybrids, can have superior cytotoxicity compared to parent molecules, exerted through multiple mechanisms, such as apoptosis and cell cycle arrest. Additionally, the in vivo analysis highlighted that benzotriazole derivatives can reduce tumor mass in a dose-dependent manner, with only a slight degree of hepatotoxicity reported in one case. However, histopathological data were generally absent or limited and based on a very limited number of in vivo studies. **Conclusions:** Overall, benzotriazole derivatives remain promising candidates for cancer treatment. However, limited mechanistic and toxicity data, as well as the moderate risk of bias identified across studies, may limit our assessment. Therefore, future studies should employ more rigorous methodologies and explore the underlying anticancer and toxicity mechanisms to fully assess the therapeutic potential of benzotriazole derivatives.

## 1. Introduction

One of the main causes of morbidity and mortality worldwide is the development of malignancies that cause millions of deaths each year [1,2]. Standard therapies, such as chemotherapy and radiotherapy, are useful treatments, but their high toxicity and tumor resistance limit their clinical use. In the last two decades, standard therapies have been replaced by targeted therapies and immunotherapies, but the latter also face tumor resistance [3,4]. Thus, these limitations are guiding ongoing research to discover alternatives with superior effectiveness and lower toxicity.

**Figure 1:**
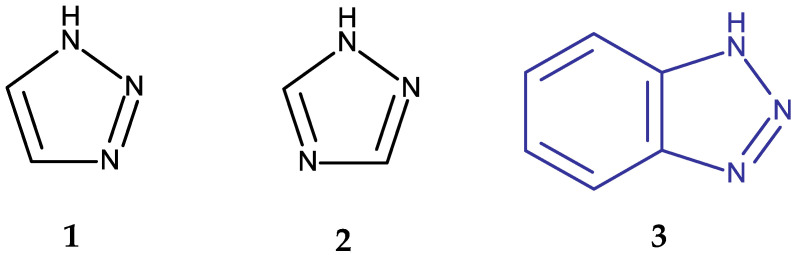
Chemical structures of 1,2,3-triazole (**1**), 1,2,4-triazole (**2**), and 1H-benzotriazole (**3**).

Heterocyclic structures have been used in medicinal chemistry as the starting point in the search for new molecules able to overcome such limitations. Some of the most studied structures in the last decade contain triazole and benzotriazole rings [5]. Triazoles are heterocyclic compounds containing three nitrogen and two carbon atoms; they are mainly found as two isomeric forms, 1,2,3-triazoles and 1,2,4-triazoles, depending on the position of the N atoms in the aromatic ring (Figure 1). Both isomers have been analyzed in numerous preclinical studies due to their ability to interact with a large number of enzymes and receptors involved in the pathogenesis of various diseases. Moreover, they have already been introduced in clinical practice as structural components of several antifungal [6], antitumor [7,8,9], antiviral [10,11], anti-anxiety [12,13], antidiabetic [14,15], and antiepileptic [16] drugs.

**Figure 2:**
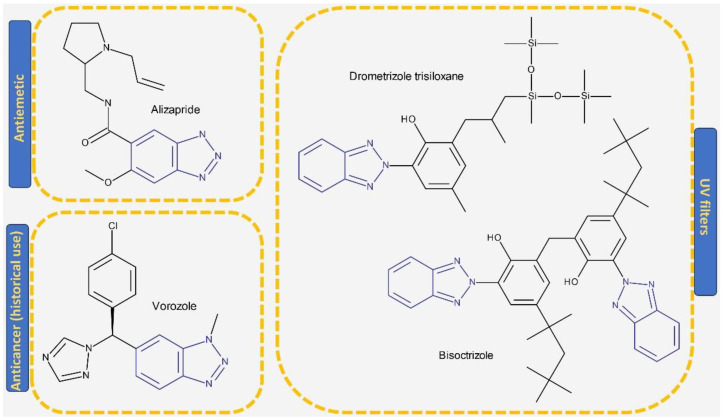
Structure of drugs and commercially available substances incorporating the benzotriazole heterocycle.

A benzotriazole is a heterocyclic compound with the molecular formula C_6_H_5_N_3_ (Figure 1) that contains one triazole ring fused with one benzene ring. It is notable for its chemical stability, versatility, and ability to interact with a wide range of biological targets [17]. Some benzotriazole derivatives, such as alizapride, a dopamine D2 receptor antagonist [18], and drometrizole trisiloxane and bisoctrizole, two UV filters found in sunscreens [19], are already commercially available (Figure 2). Another benzotriazole derivative, vorozole (Figure 2), an aromatase inhibitor used in the treatment of breast cancer [20], was discontinued due to the development of more potent aromatase inhibitors such as anastrozole and letrozole [21].

In addition to previous studies that have reviewed their synthetic methods [22] and biological activities [23], a 2022 review [24] focused on the in vitro antiproliferative mechanisms and structure–activity relationship of the benzotriazole scaffold. To our knowledge, this is the first review that systematically evaluates both in vitro and in vivo anticancer effects of benzotriazole derivatives, including a structured risk-of-bias assessment for preclinical studies. Therefore, our review aims to present an up-to-date overview of the preclinical evidence on benzotriazole derivatives as anticancer agents in order to identify gaps in the current literature that could provide future research directions.

## 2. Methodology

### 2.1. Search Strategy and Selection of Studies

We performed a search in PubMed [25], Scopus [26], and Web of Science [27] databases. The search strategy included specific terms such as benzotriazole, benzotriazole derivatives, anticancer, in vivo, in vitro models, and animal models. The search was limited to articles published in English and covered the period between January 2010 and May 2025. The reference lists were then imported into the Zotero reference manager and further exported into a Google spreadsheet for further evaluation. In the first screening phase, the titles and abstracts were independently assessed by two reviewers (G.M. and A.M.) for inclusion and exclusion criteria. In the second screening phase, the full-text articles were analyzed by two independent reviewers (G.M. and A.P.). In both screening phases, the discrepancies were resolved through discussions within the research team.

We conducted our search according to the Preferred Reporting Items for Systematic Reviews and Meta-Analyses (PRISMA) guidelines. The inclusion criteria used to screen the studies were the following: (i) benzotriazole derivatives tested in vitro or/and in vivo for anticancer effect, (ii) original full-text articles, and (iii) English language. We excluded duplicates, secondary studies, studies lacking anticancer evaluation, studies based only on in silico analysis and articles that could not be retrieved in full. The review protocol was not prospectively registered. The protocol review was retrospectively registered and is available on Zenodo [28].

### 2.2. Data Extraction

To ensure data integrity, we performed a dual data extraction with verification where G.M. independently extracted the data and A.M. verified its accuracy. Data extracted included the following: publication characteristics (name of the first author and year of publication, title, and Digital Object Identifier), compound ID, in vitro experimental model (types of cancer and cell lines, and control used)*,* in vivo experimental model (type of cancer and inoculation method, animal model, treatment, and control used), and outcomes (IC_50_/inhibition rate, tumor weight, and mechanism of action).

### 2.3. Risk of Bias Assessment

The risk of bias for the in vitro studies was evaluated by two independent reviewers (G.M. and A.P.) using the QUIN tool [29] that assesses the studies against 12 predefined criteria by employing a scoring system (2 = adequately specified, 1 = inadequately specified, 0 = not specified, and not applicable). After calculating the final score using the formula below, the risk of bias was classified as low (final score >70%), medium (50% to 70%), or high (final score < 50%).

$$Finalscore\;(\%)=\frac{total\;score}{number\;of\;criteria\;applicable}\times 50$$

The risk of bias for the in vivo studies was assessed by two independent reviewers (G.M. and A.M.) using the Systematic Review Center for Laboratory Animal Experimentation (SYRCLE RoB tool) [30], which evaluates the primary articles against six types of bias: selection, detection, performance, attrition, reporting, and other types of bias considered for this article, such as ethical approval. This resulted in three options: low risk of bias, high risk of bias, or unclear risk of bias.

## 3. Results and Discussions

**Figure 3:**
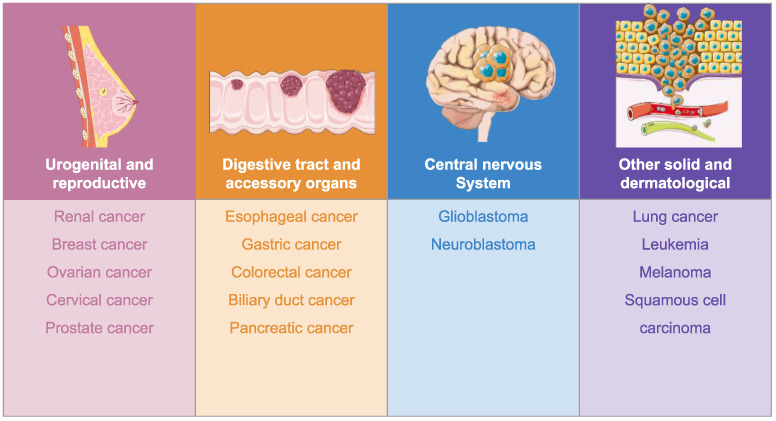
Benzotriazole derivatives in cancer. Image adapted from Servier Medical Art [31], licensed under CC BY 4.0 [32].

Our analysis revealed that benzotriazole derivatives were investigated in a preclinical setting (in vitro and in vivo) in different types of cancer (Figure 3) with promising results. We classified the derivatives in four categories, namely N-substituted derivatives, C-substituted derivatives, fused derivatives, and organometallic compounds.

**Figure 4:**
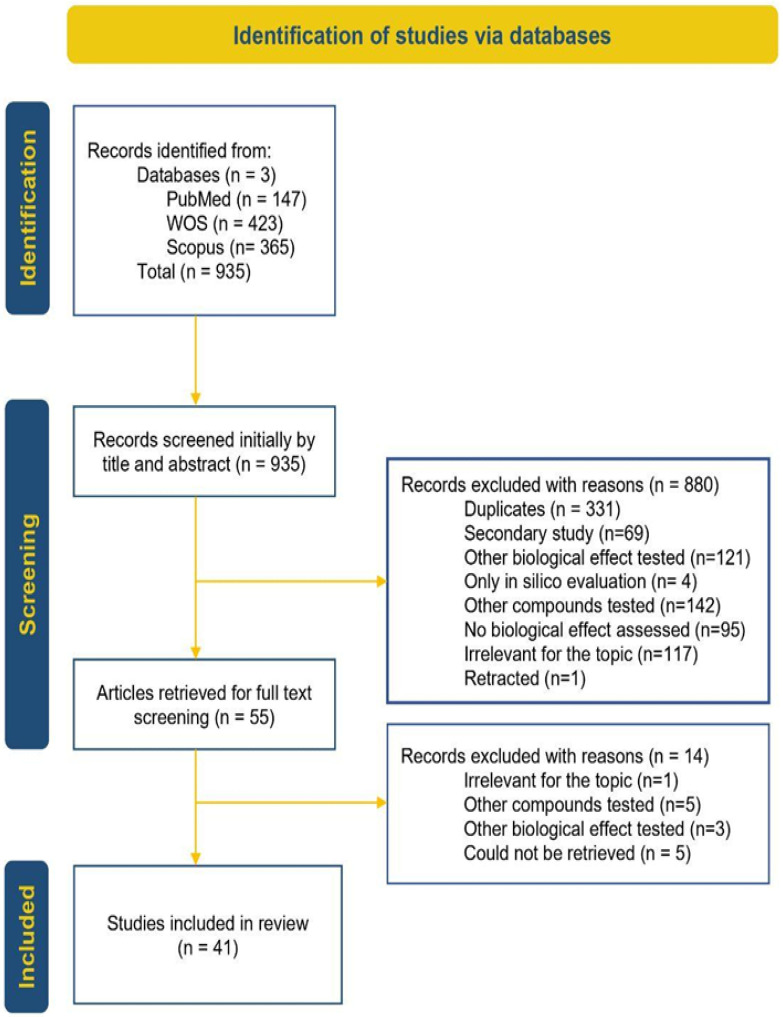
PRISMA diagram [33].

The study selection process, following PRISMA guidelines, is illustrated in Figure 4. Briefly, the 935 articles retrieved from databases underwent a two-phase screening process that ultimately resulted in 41 studies meeting the inclusion criteria and being included in the review.

### 3.1. In Vitro Evidence

#### 3.1.1. N-Substituted Benzotriazole Derivatives

The N-substitution of benzotriazole is one of the most frequently employed strategies in the development of drug candidates, with anticancer (Table 1), antibacterial [34], antiviral [35], antifungal [36], antidiabetic [37], and anticonvulsant [38] effects. The N-substitution with various alkyl or aryl substituents can be conducted at the N-1 or N-2 position, leading to high structural diversity (Table 1). In the current section, we divided the N-substituted benzotriazoles into three groups based on their structure: (i) benzotriazole-aryl derivatives, (ii) benzotriazole-alkyl-aryl derivatives, and (iii) bis-benzotriazole hybrids.

##### Benzotriazole-Aryl Derivatives

Benzotriazole-aryls are derivatives in which the benzotriazole is directly linked to an aryl fragment such as a phenyl, benzene, or other aromatic ring. The aryls can be further functionalized with halogens, electron-donating or electron-withdrawing groups that provide the molecule with improved physicochemical and biological properties.

Fabitha et al. synthesized a series of benzotriazoilphenyl-fused pyran derivatives (**ND 1–5**) and tested their effect in several cancer cell lines (Table 1). All derivatives showed modest cytotoxicity with compound **ND 1** being identified as the most active compound of the series against MCF-7 (breast cancer) cells, with the effect exerted through cell cycle arrest in the G_0_/G_1_ phase. A similar mechanism was identified for compound **ND 2**, the lead compound against HCT116 (colon cancer) cells, that induced cell cycle arrest in the G_2_/M phase [39]. The G_2_/M phase cycle arrest was also responsible for the selective cytotoxicity exerted by **ND 14**, a benzotriazolyl-imidazolyl-2,4-dichlorophenyl derivative with remarkable antiproliferative effect in MCF-7 (3.57 µM), HL-60 (0.40 µM), and HCT-116 (2.63 µM) cancer cells. The enhanced cytotoxicity of **ND 14** compared to its non-substituted (**ND 6**), monohalogenated (**ND 11–13** and **ND 15–16**) and other para-functionalized analogs (**ND 7–10** and **ND 17**) (Table 1) was attributed to the dichloro substitution on the attached phenyl ring [40]. The halogen substitution also increased the cytotoxicity of the 4-chlorophenyl-containing-benzotriazole-linked quinoline-3-carbaldehyde N-acylhydrazone (**ND 26**) and the corresponding benzotriazole-linked quinoline-3-carbaldehyde N-arylsulfonylhydrazone (**ND 34**) derivatives against DAN-G (pancreatic cancer), LCLC-103H (lung cancer), and SISO (cervical cancer) cells [41]. However, the most potent derivative of this series was a 2-pyridyl-containing-benzotriazole-linked quinoline-3-carbaldehyde hydrazone derivative (**ND 22**) with IC_50_ values below 2 μM against all tested cell lines. The benzotriazole-quinoline fragment was also found in a series of benzotriazoloquinolylin spirooxindolopyrrolizidines with cytotoxic properties against A549 (lung cancer) and HeLa S3 (cervical cancer) cells [42]; the lead candidates of this series contain two key structural elements, an ethyl-substituted aryl (**ND 41**) and a chloro-substituted aryl (**ND 44**) that significantly increased their cytotoxic effect.

In addition to the classical de novo chemical synthesis, an interesting approach is the chemical modification of natural compounds with complex structures to achieve improved cytotoxicity and selectivity [57]. Our team reported in 2022 the synthesis of three pentacyclic triterpene derivatives through the esterification of the C_28_ carboxyl group of betulinic acid (**ND 55**), oleanolic acid (**ND 56**), and ursolic acid (**ND 57**), respectively, with 1-hydroxybenzotriazole, followed by the evaluation of their antitumor activity against the A375 (melanoma) cell line. Of the three compounds, compounds **ND 55** and **ND 56** induced a dose-dependent and selective cytotoxicity against A375 cells by inhibiting mitochondrial respiration and increasing the Bax/Bcl-2 ratio [43], thus acting through apoptosis induction. Another example is the salinomycin-benzotriazole ester (**ND 58**) that induces apoptosis through a dual mechanism of cell cycle arrest in the G2/M phase and endoplasmic reticulum stress, leading to significant cytotoxic activity against MCF-7 and MDA-MB-231 breast cancer cells [44]. These studies show that benzotriazole derivatives of natural compounds exert their effect through multiple mechanisms that give them the ability to overcome drug resistance, which stands as a major limitation of the current therapies [58].

In the class of benzotriazole-linked aryl derivatives, the substitution with halogens, in particular chlorine, in the ortho or para positions increased the cytotoxic activity. This might be due to the increase in lipophilicity of the compounds, which allowed them to penetrate the cell more easily or facilitated the interaction with hydrophobic enzyme sites. Within this subclass, we observed increased efficacy particularly against breast (MCF-7 and MDA-MB-231), colorectal (HCT-116), pancreatic (DAN-G), lung (LCLC-103H and A549), and cervical (SISO, HeLa S3) cancers. In very few studies, these derivatives have demonstrated antitumor activity against leukemia (HL-60) and melanoma (A375). The effect was driven mainly by two mechanisms, the induction of apoptosis through the mitochondrial pathway and cell cycle arrest in the G2/M phase.

##### Benzotriazole-Alkyl-Aryl Derivatives

Benzotriazole-alkyl-aryl derivatives are more complex derivatives that involve the binding of the benzotriazole ring to an aryl fragment via an alkyl linker [59]; the variable length and complexity of the linker led to a large diversity of compounds that differ in terms of flexibility, polarity, and electronic distribution that ultimately may facilitate their interaction with target enzymes involved in cell survival or cell cycle progression.

Alraqa et al. synthesized three series of benzotriazole-1,2,3-triazole derivatives (**ND 59–78**) linked by a methylene group and assessed their antitumor activity in A549 and H1299 lung cancer cells, where the highest tested concentration (400 µg/mL) determined inhibition rates in the range of 75–92% [45]. Another research group used the methylene bridge to obtain benzotriazole-1,3,4-oxadiazole derivatives (**ND 79–91**) with variable cytotoxicity (87.82–4650 μg/mL) in PANC-1 (pancreatic cancer) cells [46]. Particularly, the most active derivative, **ND 82**, contained a C_5_-3,4-diethoxyphenyl substituent on the oxadiazole heterocycle that induced higher efficacy compared to the halogenated (ND 81), dimethoxy-substituted (**ND 80**), and dimethyl-substituted (**ND 79**) analogs. An even higher cytotoxicity was reported by Zhang et al. for derivatives with a more complex scaffold where the 1,3,4-oxadiazole ring was linked through a methylene bridge to the benzotriazole moiety and through a thiomethylene bridge to various substituted phenyls (**ND 92–110**) [47]. The compounds have inhibited focal adhesion kinase (FAK), involved in angiogenesis, immunosuppressive response, and cell cycle progression and survival, which is overexpressed in many malignancies [60]. However, another complex scaffold designed by Khodair et al. and containing two small methylether and thioether linker moieties in a series of glycosylated benzotriazole-phenyl-pyridine derivatives (**ND 111–126**) only led to modest cytotoxicity against MCF-7 cells through apoptosis induction [48]. The weak effect might be explained by the large molecular weight and strong molecular polarity provided by the glycosyl fragment that might hinder cell uptake, particularly since other derivatives containing smaller substituents, such as benzyl (**ND 128**) or acetyl (**ND 127**), showed superior cytotoxic effects with IC_50_ values of approximately 10 µM in HeLa (cervical cancer) cells [49].

A more complex structure of the linker through the inclusion of nitrogen atoms leads to an increased cytotoxicity. For instance, the benzotriazole-aryl derivatives (**ND 129–144**) synthesized by Anusha et al. [50] showed strong or modest cytotoxicity against the tested cell lines (Table 1). Additionally, linker ramification further improves cytotoxicity, as indicated by the assessment of a benzotriazole-acrylonitrile-linked-para-methoxyphenil derivative (**ND 145**), which exhibited IC_50_ values below 0.2 μM against tested cells (Table 1); the compound induced cell cycle arrest in the G2/M phase and apoptosis in both HeLa (cervical cancer) and PC3 (prostate cancer) cells [51].

An increased linker size, as well as the inclusion of oxygen and sulfur atoms in its structure, can further increase the cytotoxic activity of benzotriazole-alkyl linked-aryl derivatives, as shown by Kassab et al. [52] and Kumar et al. [53], who designed complex derivatives with significant in vitro cytotoxic activity. While the derivatives synthesized by Kassab et al. (**ND 146–149**) triggered apoptosis through cell cycle arrest in the G2/M phase, compound **ND 150** exhibited modest PI3K inhibitory properties and increased anti-cyclooxygenase activity, thus indicating the possibility of complementary mechanisms leading to enhanced anticancer effects [61].

The highest level of cytotoxicity was reported for benzotriazole-alkyl-aryl derivatives displaying large and branched linkers containing nitrogen, sulfur, and oxygen atoms that might favor drug–target interaction [62]; conversely, derivatives containing small chain linkers, such as methylene, induced low cytotoxic effects presumably due to an increased molecular rigidity that negatively impacts their interaction with biological targets. These derivatives showed significant cytotoxicity against lung (A549, SK-MES-1, and H-1229), breast (MCF-7), colorectal (Colo-205 and HT-29), ovarian (OVCAR-3 and A2780), and cervical (HeLa) cancer; the effect was exerted primarily through the induction of apoptosis and cell cycle in the G2/M phase, as shown in Table 1.

##### Bis-Benzotriazole Hybrids

Bis-benzotriazole hybrids are compounds containing two benzotriazole heterocycles, usually joined by an aromatic or aliphatic linker. This complex structure might allow the interaction with multiple biological targets that influence lipophilicity, cell permeability, and selectivity of certain cancer lines, thus leading to enhanced anticancer effect.

Aziz et al. designed two symmetrical bis-benzotriazole derivatives containing a diphenylmethane linker, **ND 151** [54] and **ND 152** [55], that differ only through the ether side chains grafted on the diphenylmethane core. While the more lipophilic **ND 151** derivative containing an allyloxy group showed higher cytotoxicity against the HeLa (cervical cancer) cell line, the more polar **ND 152** derivative containing a methyl acetate group had a stronger effect against the MCF-7 (breast cancer) cell line (Table 1). Although this suggests that an increased polarity might ensure an increased efficacy against MCF-7 cells, another study reported a significant cytotoxicity decrease for a more polar hybrid, a dicationic bis-benzotriazolium salt containing a substituted diphenyl-methylene bridge linker (**ND 153**) [56], thus indicating that **NF 153** falls outside the optimal lipophilicity window required for passive uptake in MCF-7 cells [63]. Such results might mirror the structural differences between different cell types that cause differences in the cellular uptake and metabolic processes, ultimately influencing the quantity of active substance that reaches its target [64,65]. Although the number of N-substituted bis-benzotriazoles studied as anticancer agents is limited, the underlying in vitro mechanism of the most promising hybrids should be further explored.

Bis-benzotriazole derivatives were tested mainly in cervical (HeLa) and breast cancer (MCF-7) cell lines where the effect was dependent on the polarity of the derivatives. These effects could be determined by both an increased affinity for a target sites or by an increase in intracellular uptake. Future studies might expand the research of these derivatives in other cancer lines and explore the molecular targets, as the current studies did not provide any mechanistic analysis.

#### 3.1.2. C-Substituted Derivatives of Benzotriazole

According to our analysis, the most investigated C-substituted benzotriazole derivative was 4,5,6,7-tetrabromobenzotriazole (**CD 1**) (Table 2) due to its ability to inhibit CK-2, a protein involved in the regulation of several cancer-related proteins (HDAC1 and HDAC2, NF-κB, and p53) and a potential prognostic marker for several cancers, including leukemia, breast, and colon cancer [66].

Trembley et al. investigated the **CD 1** inhibitory effect against CK-2 in androgen-sensitive and insensitive prostate cancer cell lines (LNCap and PC-3). **CD 1** reduced the expression of the androgen receptor in the PC-3 cell line and decreased the concentration of the NF*κ*B p65 subunit, a pro-survival protein, in both cell lines, leading to a subsequent reduction in proliferation and viability, and thus demonstrating an androgen-independent anticancer effect [67]. The inhibition of CK-2 was also confirmed in WiDr colon cancer cells [68], where **CD 1** induces DNA damage and interferes with the phosphorylation of XRCC1, a key protein in the base excision repair mechanism [82], thus increasing cell apoptosis. Another interesting mechanism was uncovered in chronic lymphocytic leukemia, where **CD 1** restored PTEN activity in CLL cells, thereby inhibiting the PI3K/Akt pathway and leading to a dose-dependent apoptosis induction [69].

**CD 1** can serve as a scaffold for further derivatizations as shown by Chojnacka et al. [70], who initially obtained an N_2_-chloropropyl intermediate that was further alkylated using various 5-aryltetrazoles (**CD 2–6**); the resulting compounds exhibited increased cytotoxicity, particularly against MCF-7 cells. However, the underlying mechanism involved is CK-2-independent, as none of the derivatives has inhibited human recombinant CK2α in vitro. The **CD 1** triazolylalkyl-substituted derivatives where the triazole was substituted with oxygenated groups such as hydroxyl, carboxyl, or ethyl ester (**CD 7–11**), showed modest activity against MCF-7 cells [71] compared to derivatives containing aminoalkyltriazolyls (**CD 12–14**) [72] that exhibited similar IC_50_ values to the 5-aryltetrazole derivatives (**CD 2–6**) [70]. Triazole substitution with larger radicals, such as the proxyphylline-benzotriazole hybrid exhibiting a 1,2,3-triazole linker (**CD 15**), also leads to low cytotoxicity against MCF-7 cells [73]. Additionally, N_2_ substitution using aminoalkyltriazolyl groups (**CD 13–14**) resulted in more potent derivatives compared to N_1_ substitutions (**CD 12**), regardless of the tested cell line [72], thus suggesting that the N_2_ substitution may be a more oriented strategy for the synthesis of anticancer agents. The N_2_ aliphatic substitution of **CD 1** can also lead to active compounds, as shown for the N_2_-carbohydrazidomethyl derivative (**CD 16**) synthesized by El-Kardocy et al. [74] that exhibited comparable cytotoxicity against MCF-7 cells to the most active 5-aryltetrazoles (**CD 3–6**) [70] (Table 2).

Apart from **CD 1**, the benzotriazole C-substitution with halogens was also used to develop other anticancer candidates such as the 3-(1-(7-bromobenzotriazolyl))-nor-β-lapachones (**CD 17–21**) that showed IC_50_ values of approximately 2 μM against MCF-7 (breast cancer), HepG2 (hepatocellular cancer), and A549 (lung cancer) cells [75]. Other examples are the mono- and bis-5,6-dichloro-benzotriazole derivatives (**CD 22–25**) obtained by Ibba et al. [76] who noted that the anticancer activity was only recorded for the chloride-containing benzotriazole derivatives such as **CD 22**, a bis-5,6-dichloro-benzotriazole derivative linked through a propane-1,3-diyl diamide spacer, which was considered the lead compound; after further evaluation, it showed an apoptotic effect in SK-MES 1 (lung cancer) cells. In a series of benzotriazole–ethynyl–pyridyl hybrids (**CD 26–35**), the chloride substitution of the phenyl ring in benzotriazole also increased cytotoxicity against VX2 (squamous cell carcinoma), A549 (lung cancer), MGC-803, and MKN45 (gastric cancer) cells [77]. Compound **CD 34**, displaying a N_1_-(3-chloropyridin-2-yl)ethynyl fragment and a C_5_-chloro substituent grafted onto the benzotriazole ring, was considered the lead candidate of the series, with an IC_50_ value below 5.5 μM across all tested cell lines; its biological effects were superior to those exhibited by both the N_2_-substituted analogs (**CD 30–33**) and the N_1_-substituted analogs bearing a C_6_-chloro moiety. However, halogen substitution does not always lead to increased cytotoxicity as shown by Wang et al. [78] who synthesized unsubstituted (**CD 37**), fluoro-substituted (**CD 36**), and chloro-substituted (**CD 38**) naphthalimide-benzotriazole conjugates. While the chloro-substituted derivative only showed comparable cytotoxicity against MDA-MB-231 (breast cancer) cells with the unsubstituted derivative, considered the lead compound of the series, the fluoro-derivative performed even more modestly against all tested cell lines. These results suggest that, when considering C-halogen substitution of benzotriazoles as a strategy to increase their cytotoxicity, chloro substitution may be more effective than other halogens. Moreover, introducing more than one type of halogen atom could further enhance the effect, as shown by the **CD 1** halogenated derivatives.

Another strategy employed the C-derivatization of the benzotriazole scaffold with substituted pyrimidines (**CD 39–54**) to obtain multikinase inhibitors for cancer treatment; this strategy yielded compound **CD 53** with nanomolar IC_50_ values (9–41 nM) against four types of cancer cells [79], and which inhibits the activity of CDK and FLT kinases involved in tumor development and progression [83,84]. Alternatively, Entezari et al. synthesized a series of compounds by modifying carboxylated multilayer carbon nanotubes (MWNT–COOH), ultimately obtaining new functional benzotriazole derivatives bearing a sulphonic acid group at the C_6_ position of the benzotriazole ring (**CD 55–57**); these derivatives exerted superior cytotoxic effects against the MKN-45 (gastric cancer) and SW742 (colon cancer) cell lines to doxorubicin and cisplatin that were used as positive controls [80].

In the C-derivatives class, some derivatizations were found to be beneficial both for increasing the antitumor effect and selectivity against certain molecular targets, such as casein kinase 2 (CK2), an important target for **CD 1** and its derivatives, as well as other targets involved in apoptosis and cell cycle arrest. For instance, the substitution with pyrimidine or halogens, such as bromine, was found to be preferable in increasing the cytotoxicity in colon (WiDr), breast (MCF-7 and MDA-MB-231), hepatic (Hep G2), ovarian (SKOV-3), and cervical (SiHa) cancer.

#### 3.1.3. Fused Benzotriazole Derivatives

A fused derivative of thiadiazolobenzotriazole (**FD 1**) embedded in peptide cRGD surface-modified nanoparticles was studied as a theranostic agent against glioblastoma and demonstrated high photothermal efficacy under the NIR-II light. This strategy uses thiadiazolobenzotriazole as a strong electron acceptor from dithienopyrrole and facilitates heat generation under the NIR light, thereby triggering cytotoxic effects through necrosis and apoptosis [81]. This is the only study included in our analysis that investigated a fused derivative of benzotriazole, providing a promising strategy that should be further explored in order to develop theranostic agents.

#### 3.1.4. Organometallic Compounds Containing Benzotriazole

Organometallic compounds have been explored as potential anticancer drugs since the introduction of cisplatin (cis-diamminedichloroplatinum(II)), a molecule that remains highly relevant in modern cancer treatment [85,86]. Although such compounds affect multiple pathways involved in cell death, such as apoptosis, necrosis, autophagy, pyroptosis, and ferroptosis, challenges like tumor resistance and targeted action still need suitable approaches [81]. The most studied types of organometallic complexes are metallocenes, metal-arene, and metal-carbonyl [87]; however, increased use of N-containing heterocycles, such as benzotriazoles, as ligands has also been reported (Table 3).

Onar et al. reported the synthesis of a series of Pd (II) and Ru (II) complexes using several benzotriazole-N-substituted carbenes as ligands (**OM 1–8**) and assessed their anticancer effect in breast (MCF-7) and colon (Caco-2) cancer cells; the Ru (II) complexes (**OM 5–8**) showed higher cytotoxicity compared to the Pd (II) complexes (**OM 1–4**) and similar to the cisplatin used as a positive standard [87].

Stamou et al. reported the synthesis and antiproliferative effect of a Sn (IV) complex (**OM 9**) bearing a 1-methylbenzotriazole moiety in MDA-MB-231 breast cancer cells with an IC_50_ = 20 µM [88]. Moreover, El-Asmy et al. synthesized a large series of organometallic complexes containing a 2-(2′-hydroxy-5′-methylphenyl)-benzotriazole fragment (**OM 10–18**) of which the complexes with Ag (I) (**OM 16–17**), Pt (II) (**OM 13, OM 15**), Pd (II) (**OM 12, OM 14**), and Rh (II) (**OM 18**), respectively, showed an even higher cytotoxic efficacy against MDA-MB-231 cells with IC_50_ values ranging between 1.37 and 18.89 µM. Additionally, the same complexes showed strong activity against the OVCAR-8 cell line with IC_50_ values ranging from 1.75 to 23.54 µM, while Zn (II) (**OM 10–11**) complexes exerted little effect in both cell lines [89]. The superior effects of Pd (II) (**OM 20**) and Pt (II) (**OM 21)** compared to Zn (II) complexes (**OM 19**) in breast cancer, both in terms of cytotoxicity and angiogenesis inhibition, were also reported when the N-(2-thiazolyl)-1H-benzotriazole-1-carbothioamide fragment was used as a ligand [90].

Another ligand containing the benzotriazole heterocycle is the 1-((2-(pyridin-2-yl)-1-benzoimidazol-1-yl)methyl)-1H-benzotriazole used by Zhao et al. to form Zn (II) (**OM 22**) [91], Cu (II) (**OM 23**), and Co (II) (**OM 24**) complexes [92]. The resulting complexes showed a modest in vitro anticancer effect (IC_50_ > 30 µM) against the tested cell lines (Table 3) through apoptosis induction and cell cycle arrest in G0/G1 (**OM 22**) and G2/M (**OM 23**). Other Cu (II) complex with 1((2-(pyridine-3-yl)-1H-benzoimidazol-1-yl) methyl)-1H-benzotriazole (**OM 25**) induced apoptosis and cell cycle arrest in the G1 phase in HeLa (cervical cancer) and SGC-7901 (gastric cancer) cells with superior cytotoxicity observed after prolonged exposure (72 h vs. 48 h) (Table 3) [93]. However, when the structural isomer 1-((2-(pyridin-2-yl)-1-benzoimidazol-1-yl)methyl)-1H-benzotriazole was used in other Cu (II) complexes (**OM 26–27**), its anticancer activity was inferior to its non-benzotriazole analogs in breast (MCF-7), esophageal (EC109), neuroblastoma (SH-SY5Y), and biliary duct cancer (QBC939) cells with the strongest effect reported against the EC109 cell line (IC_50_ = 14.05–28.75 µM) [94], thus indicating that structural isomer ligands might be employed for targeting different cell lines. Future studies regarding the anticancer mechanisms of such complexes might expand our understanding of how using certain ligands could target different cancer cell types.

In the organometallic series, benzotriazole was used as a ligand to potentiate the therapeutic efficacy of the metal associated. In this class, it can be observed that the metal used to form the organometallic complex has a great impact on the overall toxicity, such as the Pd(II) and Pt(II) derivatives, which had a superior effect compared to the Zn(II) complex. These complexes exhibited significant antitumor activity mainly in breast (MCF-7 and MDA-MB-231), colorectal (HCT116 and Caco-2) and gastric cancer (BGC-823 and SGC-7901). Future studies might investigate the cytotoxicity of benzotriazole complexes with other metals with cytotoxic properties, such as rare earth metals [95].

#### 3.1.5. Risk of Bias Assessment (QUIN Tool)

The QUIN tool, comprising 12 assessment criteria, was used to assess the in vitro risk of bias. Each criterion was assessed with a score between 0 and 2 depending on the compliance degree and the final score was calculated according to the formula recommended by the authors [29]. The percentage of the final score ranged from 54.17% to 66.67% with an average value of 61.48%. Thus, according to the thresholds defined in QUIN Tools, all studies fall into the “Medium risk of bias” category, suggesting that the analysis method was clear and consistent. Analyzing the scores of the 12 criteria in more detail, we noticed certain criteria with maximum scores, others with moderate scores, and some that were not scored at all. Among the criteria with maximum scores (2), are clearly stated aims/objectives, a detailed explanation of methodology, a method of measurement of the outcome, and presentation of results. Other criteria such as a detailed explanation of the sampling technique (1.98) and details of the comparison group (1.88), had an arithmetic mean close to two (the maximum score). The results obtained for these criteria indicate both procedural transparency and accurate reporting of the experimental stages in most studies. Statistical analysis, outcome assessor details, and a detailed explanation of sample size calculation are the criteria that obtained a final average score between 0.75 and 1.20, suggesting that statistical analyses, details justifying the sample size, and validation of results by evaluators were reported partially or incompletely. The criteria where the score was zero (operator details, randomization, and blinding) indicate that randomization or blinding procedures are not applied or reported in in vitro studies, thus suggesting that it is quite difficult to correct or prevent systematic errors [96]. Furthermore, the absence of information about who performed the experiments suggests a high risk of uncontrolled and subjective inter-operator variability [97,98]. Other authors who used this scale to assess the risk of bias in in vitro studies obtained similar results for operator details, randomization, blinding, detailed explanation of sample size calculation, and outcome assessor details, where the mean was around one [99,100,101,102]. These results suggest that future studies must improve experimental standardization, randomization, and transparent reporting practices, aspects that could significantly improve the quality and credibility of in vitro studies.

### 3.2. In Vivo Evidence

#### 3.2.1. Qualitative Synthesis

The promising results obtained in vitro are not always confirmed or fully translated into in vivo models due to vascularization, extensive metabolism, and immune response, depending on the model’s complexity [103,104]; however, they provide a starting point for elucidating the mechanism of action underlying the therapeutic effect. The available data suggest that in vivo benzotriazole derivatives exert their anticancer effect through several mechanisms, such as kinase inhibition, induction of apoptosis, and inhibition of angiogenesis, which ultimately lead to tumor reduction and necrosis (Table 4). Nonetheless, only a limited number of studies employed an in vivo assessment and further studies are needed to confirm the hypothesis.

The C-substituted derivatives tested in animal models showed CK-2 or multikinase inhibition leading to tumor reduction in prostate (22Rv1), colon (WiDr), and squamous cell carcinoma (SiHa) models. **CD 1** inhibited CK-2 in colon [68] and prostate xenografts [67]; moreover, CK-2 inhibition reduced androgen receptor expression and decreased NFκB p65 total protein levels, markers associated with cell survival and prostate cancer progression (Table 4) [105,106]. Significant tumor reduction was also reported for the pyrimidine-benzotriazole derivative (**CD 53**) [79] and benzotriazole-ꞵ-lapachone derivative (**CD 21**) [75], although the mechanisms responsible for such an effect were not investigated (Table 4).

The fused derivative used by He et al. (**FD 1**) [81] in the photothermal therapy of glioblastoma decreased **CD31**, a marker for angiogenesis [107], and Ki67-positive cells, a marker for cell proliferation [108], thus suggesting that the ablation therapy might trigger multiple pathways leading to tumor reduction. Tumor reduction was also reported in the presence of the Cu (II)-organometallic complex (**OM 25**) [93] as a result of the inhibition of the cyclinD1/cdk4 pathway and subsequent G1 cell cycle arrest.

Regarding the toxicity of these compounds in vivo, the available data are inadequate to draw a reliable conclusion at the moment, as the histopathological analyses are missing in the majority of the studies, with only one study reporting some degree of hepatotoxicity for the **OM 25** organometallic complex [93]. Future studies should include histopathological analysis to validate the lack of toxicity for these compounds. Future preclinical studies should adopt standardized tumor models, dosing schemes, and toxicity endpoints, including histopathology and organ-specific biomarkers to allow comparison across derivatives and facilitate translation.

#### 3.2.2. Risk of Bias Assessment (SYRCLE Tool)

**Figure 5:**
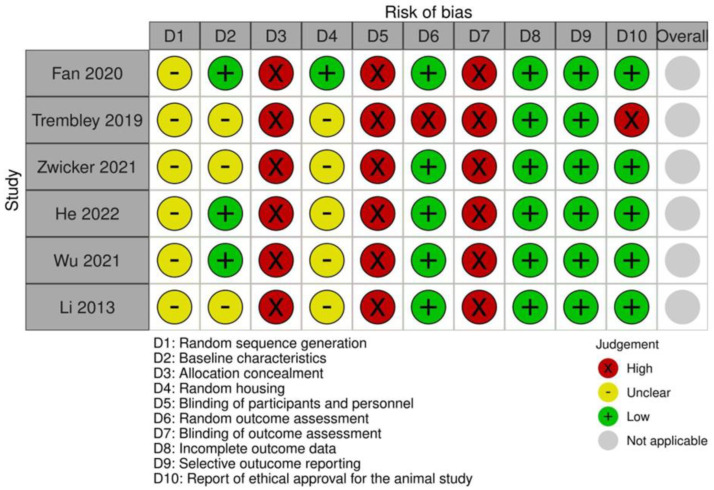
Risk of bias assessment according to SYRCLE criteria. References: Fan 2020 [79], Trembley 2019 [67], Zwicker 2021 [68], He 2022 [81], Wu 2021 [75] and Li 2013 [93].

The risk of bias assessment is shown in Figure 5. Regarding selection bias, no article reported random sequence generation. Three articles (50%) described the baseline characteristics of the animals used, considering animal type, age, and sex, but no study reported allocation concealment. Concerning performance bias, only one study (16.66%) reported random animal housing, while no study mentioned whether personnel were blinded regarding the treatment administered. For detection bias, five studies (83.33%) reported random assessment of outcomes, but none described blinding the evaluator to the treatment groups (benzotriazole-treated versus control). All studies (100%) reported data from all animals included and reported all expected outcomes. Regarding other biases, five studies (83.33%) reported having ethical approval. Our analysis might be limited because no study reported allocation concealment or blinding of evaluators. This lack may result from the small number of researchers involved or the homogeneity of the animals specifically selected for identical genetic background, same diets, and housing conditions [109]. Another significant limitation is the failure to report on the randomization method used, an aspect associated with reduced reproducibility [110,111], which could lead to an overestimated effect of the benzotriazole derivatives in animal models of cancer. [112,113]. Additionally, our review is limited by the small number of studies on benzotriazole derivatives in animals. Since the risk of bias analysis shows no study met all criteria, future studies should adopt more rigorous methodologies.

## 4. Conclusions

Benzotriazole derivatives have been developed by several researchers due to the chemical versatility of the heterocycle and its interaction with a plethora of biological targets, leading to promising anticancer effects. The use of benzotriazole as a chemical scaffold for synthetic and semi-synthetic derivatizations leads to promising cytotoxicity and might enhance other synergistic biological effects of the developed anticancer drugs.

Our study revealed that the N-substituted class was cytotoxic mainly against breast, lung, colorectal, and cervical cancers, while the C-substituted class had significant antitumor activity predominantly against leukemia, breast, and lung cancer. Moreover, the organometallic derivatives showed cytotoxic activity predominantly against breast, colorectal, and gastric cancers. Also, the class of fused benzotriazole derivatives shows activity only against glioblastoma, being the only type of cancer investigated for these derivatives.

The benzotriazole derivatives can induce apoptosis through the intrinsic pathway and provoke cell cycle arrest in certain phases such as, G0/G1, S, and G2/M, thus they might be able to increase therapeutic efficiency by overcoming treatment resistance. In terms of chemical structure, our analysis shows that any chemical modification directly influences the cytotoxic activity of the derivatives. For example, substitution with one or more halogens increases cytotoxicity, as shown in the case of 4,5,6,7-tetrabromobenzotriazoles (**CD 52**) derivatives. In the case of benzotriazole-alkyl-aryl derivatives, longer and more complex linkers display higher cytotoxicity, presumably due to stronger drug-target interaction. Moreover, an interesting strategy that led to significant cytotoxic effects was the synthesis of benzotriazole-bearing analogs of natural compounds such as betulinic acid, oleanolic acid, and salinomycin.

The in vivo analysis showed that the benzotriazole derivatives were able to exert anti-tumor effects in xenograft models by inducing apoptosis through the inhibition of the cyclin D1/CDK4 pathway, inhibiting angiogenesis by down-regulating **CD31** levels and kinase inhibition through the down-regulation of the casein kinase II (CK2). The histopathological assessment was reported only in a study that showed some degree of hepatotoxicity of the organometallic complex tested, while the other studies failed to report or were limited to basic measures such as body weight. The major limitations of our in vivo analysis reside in the low number of studies available for assessment, the lack of toxicity data and the shortcomings identified by the risk of bias assessment, as no study reported allocation concealment and randomization.

From a medicinal chemistry perspective, the benzotriazole emerges as a versatile anticancer scaffold whose antitumor activity can be finely tuned through halogenation, linker engineering, and hybridization with natural or organometallic pharmacophores. Future studies must employ more rigorously designed methodologies, present a more in-depth analysis of the underlying molecular mechanisms of the reported biological effects and assess more thoroughly the toxicity of the derivatized benzotriazoles. Overall, while the current evidence on the therapeutic potential of benzotriazole derivatives is promising, further research is required for clinical translation.

## Figures and Tables

**Table 1:** N-substituted derivatives of a benzotriazole (N/A—no data available within the article; stereochemistry is shown only when reported in the original studies; the original compound IDs are given in parentheses; ↑ indicates induction or up-regulation; and ↓ indicates inhibition or down-regulation).

Chemical Structure		Compound ID	IC_50_ (μM or μg/mL), Inhibition Rate (%)	Mechanism of Action	Reference
Core Structure	Substituents				
**Direct benzotriazole-aryl derivatives**					
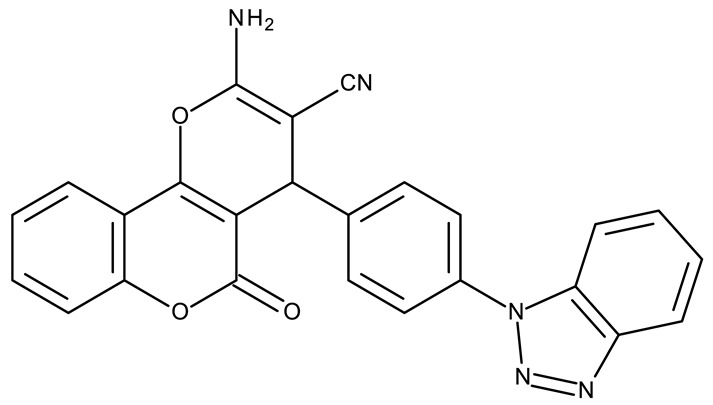	-	**ND 1** (6e)	MCF-7: 12.47 μM A549, HCT116: >50 μM	↑cell cycle arrest (G0/G1, S and G2/M)	[39]
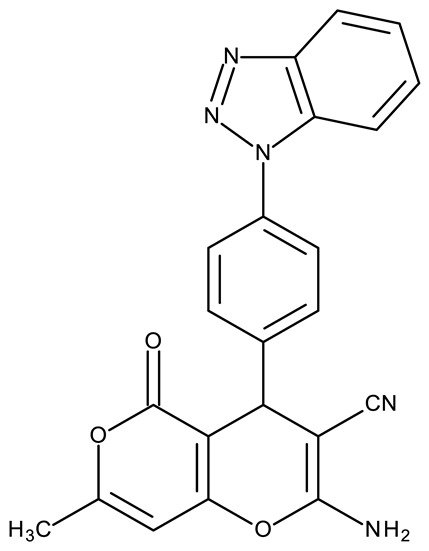	-	**ND 2** (8e)	HCT116: 21.43 μM MCF-7, A549: >50 μM		
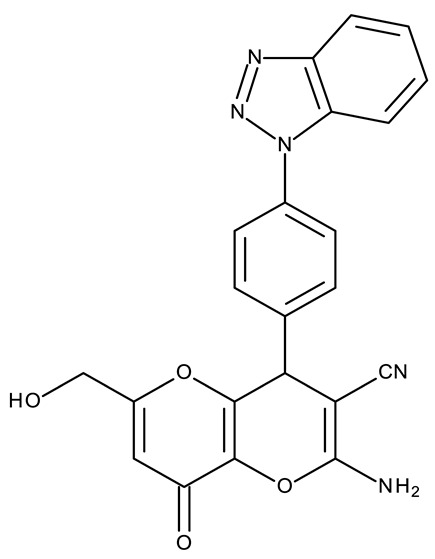	-	**ND 3** (10e)	MCF-7, A549, HCT116: >50 μM		
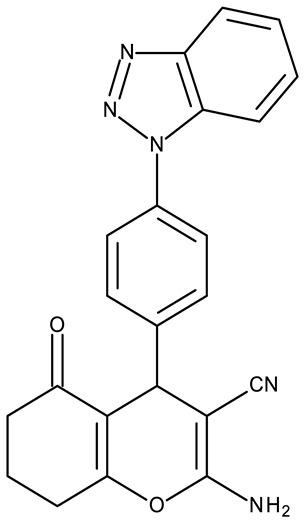	-	**ND 4** (12e)	MCF-7, A549, HCT116: >50 μM		
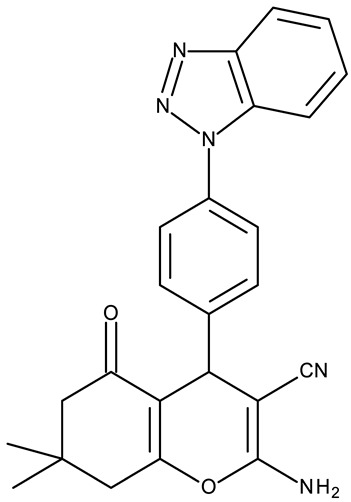	-	**ND 5** (14e)	MCF-7, A549, HCT116: >50 μM		
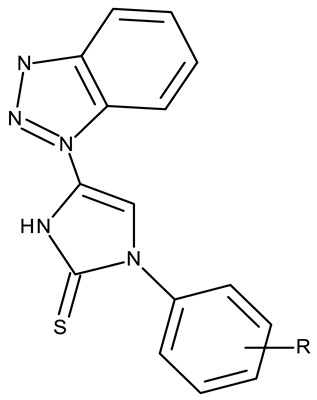	R = H (**ND 6**) R = 4-methyl (**ND 7**) R = 4-ethyl (**ND 8**) R = 4-methoxy (**ND 9**) R = 4-hydroxyl (**ND 10**) R = 4-Cl (**ND 11**) R = 2-Cl (**ND 12**) R = 3-Cl (**ND 13**) R = 2,4-(Cl)_2_ (**ND 14**) R = 2-F (**ND 15**) R = 4-Br (**ND 16**) R = 4-sulphone-amide (**ND 17**)	**ND 6–17** (BI1–12)	MCF-7: 2.29–38.2 μM HL-60: 0.4–37.1 μM HCT-116: 1.51–17.5 μM	↑apoptosis by ↑PARP cleavage, ↑BAX and ↓Bcl-2; ↑cell cycle arrest (G2/M)	[40]
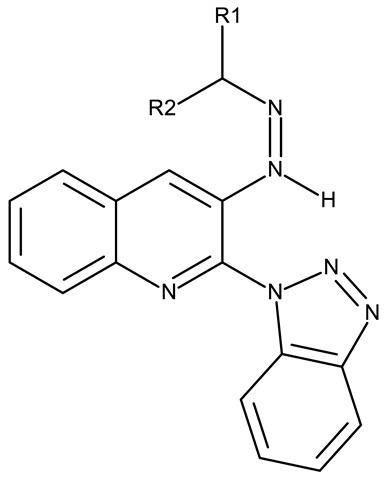	R1 = H, R2 = H (**ND 18**) R1 = methyl, R2 = methyl, (**ND 19**) R1 = H, R2 = phenyl (**ND 20**) R1 = H, R2 = 2-hydroxyethyl (**ND 21**) R1 = H, R2 = 2-pyridyl (**ND 22**)	**ND 18–22** (5a–e)	DAN-G: 1.35–6.38 μM LCLC-103H: 1.23–6.29 μM SISO: 1.49–6.23 μM	N/A	[41]
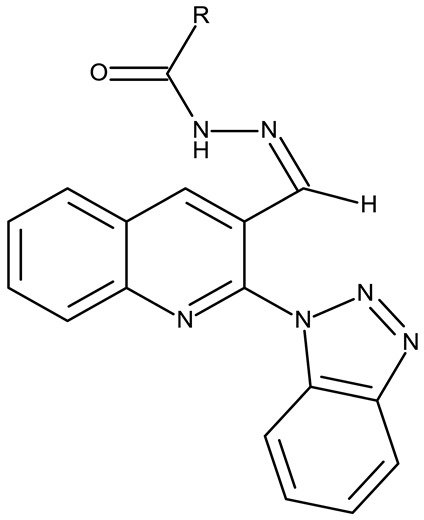	R = phenyl (**ND 23**) R = 4-methylphenyl (**ND 24**) R = 4-methoxyphenyl (**ND 25**) R = 4-chlorophenyl (**ND 26**) R = 4-fluorophenyl (**ND 27**) R = 2-furyl (**ND 28**) R = 2-thienyl (**ND 29**) R = cyclopentyl (**ND 30**)	**ND 23–30** (7a–h)	DAN-G: 0.6–56.3 μM LCLC-103H: 3.6–51.1 μM SISO: 0.2–53.8 μM		
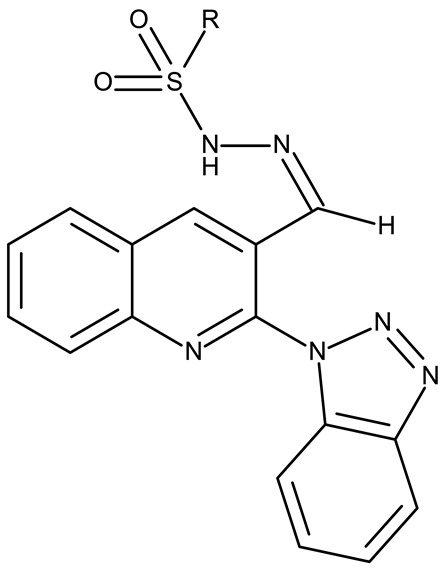	R = phenyl (**ND 31**) R = 4 methylphenyl (**ND 32**) R = 4-methoxyphenyl (**ND 33**) R = 4-chlorophenyl (**ND 34**) R = 4-fluorophenyl (**ND 35**) R = 2,4,6-trimethylphenyl (**ND 36)** R = 4-tert-butylphenyl (**ND 37**) R = 2-naphthyl (**ND 38**)	**ND 31–38** (9a–h)	DAN-G: 26.1–82.1 μM LCLC-103H: 8.1–37.5 μM SISO: 0.6–34.4 μM		
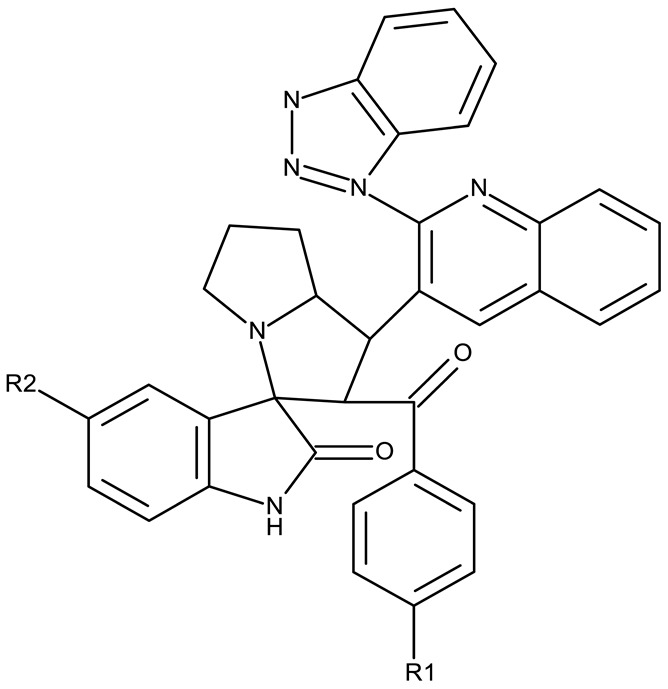	R1 = H, R2 = H (**ND 39**) R1 = H, R2 = methyl (**ND 40**) R1 = H, R2 = ethyl (**ND 41**) R1 = H, R2 = methoxy (**ND 42**) R1 = H, R2 = F (**ND 43**) R1 = H, R2 = Cl (**ND 44**) R1 = H, R2 = Br (**ND 45**) R1 = Cl, R2 = H (**ND 46**) R1 = Cl, R2 = methyl (**ND 47**) R1 = Cl, R2 = ethyl (**ND 48**) R1 = Cl, R2 = methoxy (**ND 49**) R1 = Cl, R2 = F (**ND 50**) R1 = Cl, R2 = Cl (**ND 51**) R1 = Cl, R2 = Br (**ND 52**) R1 = Br, R2 = H (**ND 53**) R1 = Br, R2 = methoxy (**ND 54**)	**ND 39–54** (4a–p)	A549: 9.81–>100 μM HeLa S3: 20.4–>100 μM	N/A	[42]
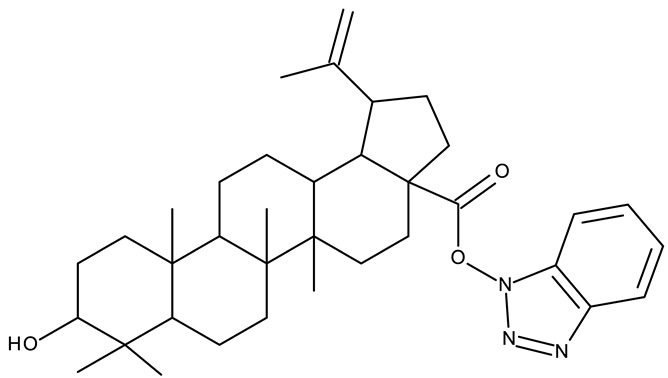	-	**ND 55–57** (1–3)	A375: 12.6–37.5%	↑apoptosis (intrinsic pathway)	[43]
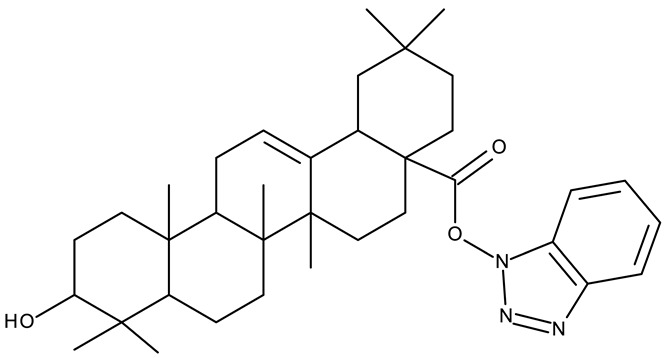	-				
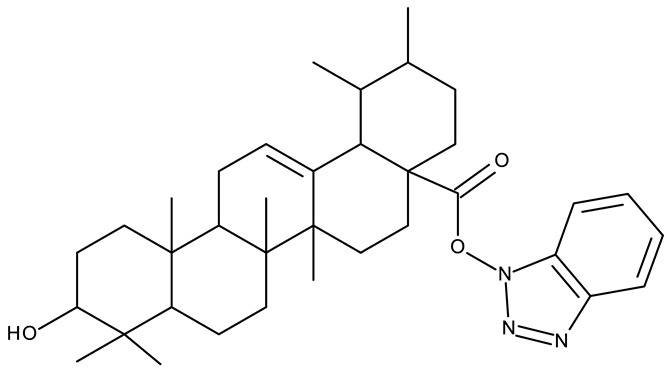	-				
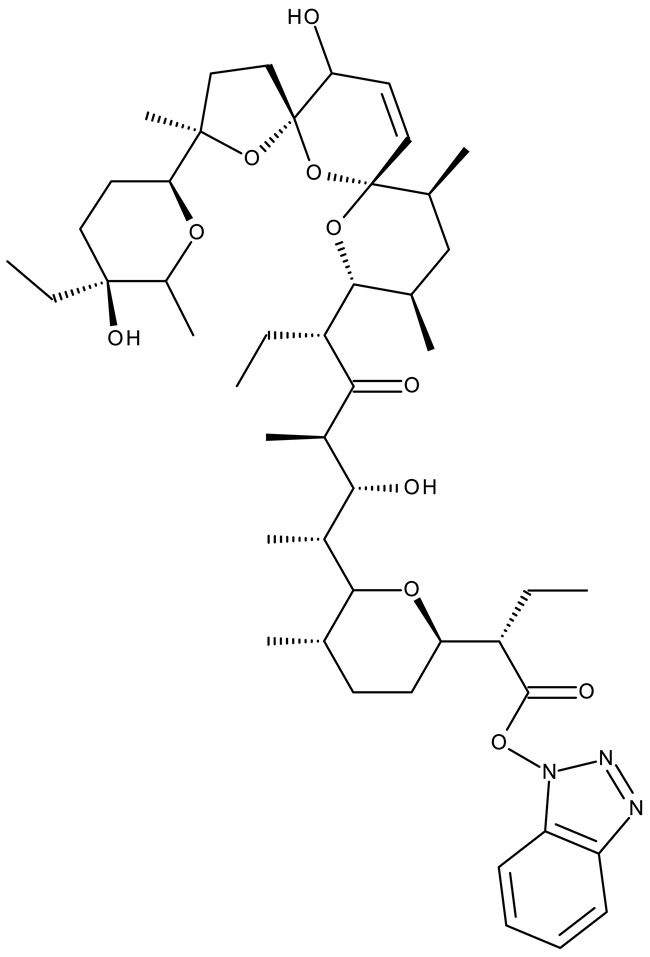	-	**ND 58** (7)	MCF-7: 4.1 μM MDA-MB-231: 2.6 μM	↑apoptosis (intrinsic pathway); ↑cell cycle arrest (G2/M); ↑p53; ↑p-eIF2α; ↑IRE1α ↑γH2AX and ↑8-oxoG	[44]
**Benzotriazole-alkyl-aryl derivatives**					
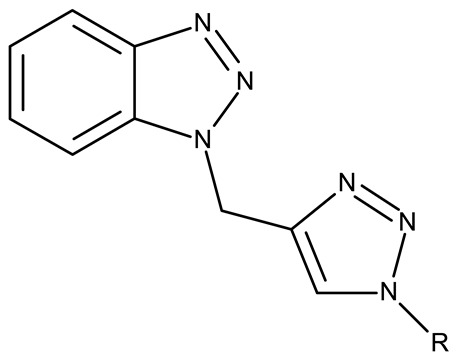	R = 4-nitrophenyl (**ND 59**) R = 3,4-dichlorophenyl (**ND 60**) R = 4-acetylphenyl (**ND 61**) R = 4-carboxyphenyl (**ND 62**) R = 4-ethoxycarbonylphenyl (**ND 63**) R = benzyl (**ND 64**) R = phenethoxy (**ND 65**) R = ethoxycarbonylethyl (**ND 66**) R = 4-methoxyphenylacetyl (**ND 67**) R = 4-methylphenylacetyl (**ND 68**) R = N-benzylacetamide (**ND 69**) R = N-(4-methoxybenzyl)acetamide (**ND 70**) R = N-(4-fluorobenzyl)acetamide (**ND 71**) R = N-(4-chlorobenzyl)acetamide (**ND 72**) R = N-(4-fluorophenyl)acetamide (**ND 73**) R = N-(2-fluoro-4-iodophenyl)acetamide (**ND 74**) R = N-(2,4,5-trifluorophenyl)acetamide (**ND 75**) R = N-(4-carboxyphenyl)acetamide (**ND 76**) R = N-(4-nitrophenyl)acetamide (**ND 77**) R = N-(4-iodophenyl)acetamide (**ND 78**)	**ND 59–78** (4a–e, 6a–e, 8a–j)	A549: 78–89% H-1229: 70–92%	N/A	[45]
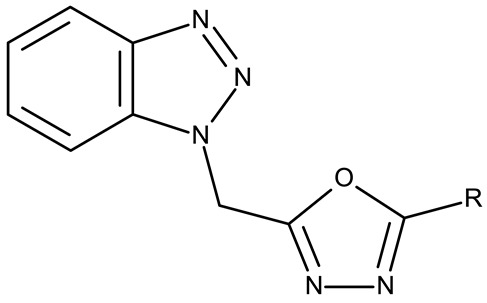	R = 3,4-dimethylphenyl (**ND 79**) R = 3,4-dimethoxyphenyl (**ND 80**) R = 4-trifluoromethylphenyl (**ND 81**) R = 3,4-diethoxyphenyl (**ND 82**) R = 2-methylphenyl (**ND 83**) R = 3,5-dimethoxyphenyl (**ND 84**) R = 2-naphthyl (**ND 85**) R = 3-chloro-4-nitrophenyl (**ND 86**) R = 6-quinolyl (**ND 87**) R = 4-hydroxy-3-methoxyphenyl (**ND 88**) R = 4-hydroxyphenyl (**ND 89**) R = 4-sulfamoylphenyl (**ND 90**) R = 4-ethylphenyl (**ND 91**)	**ND 79–91** (4a–m)	PANC-1: 87.82–4650 μg/mL	N/A	[46]
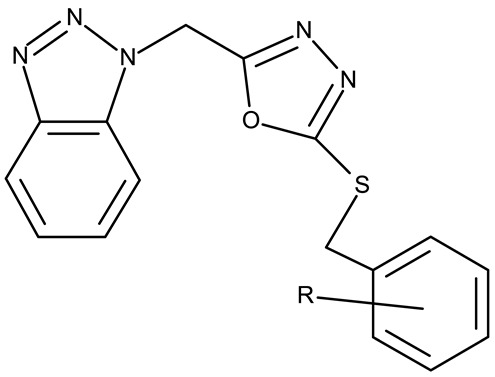	R = 2-F (**ND 92**) R = 2-Cl (**ND 93**) R = 2-Br (**ND 94**) R = 2-methyl (**ND 95**) R = 2-methoxy (**ND 96**) R = 2-nitro (**ND 97**) R = 3-F (**ND 98**) R = 3-Cl (**ND 99**) R = 3-Br (**ND 100**) R = 3-methyl (**ND 101**) R = 3-methoxy (**ND 102**) R = 3-nitro (**ND 103**) R = 4-F (**ND 104**) R = 4-Br (**ND 105**) R = 4-methyl (**ND 106**) R = 4-methoxy (**ND 107**) R = 4-nitro (**ND 108**) R = 3,4-dichloro (**ND 109**) R = 3,4-difluoro (**ND 110**)	**ND 92–110** (4–22)	MCF-7: 5.68–45.16 μM HT-29: 10.21–42.30 μM	↑apoptosis ↓FAK	[47]
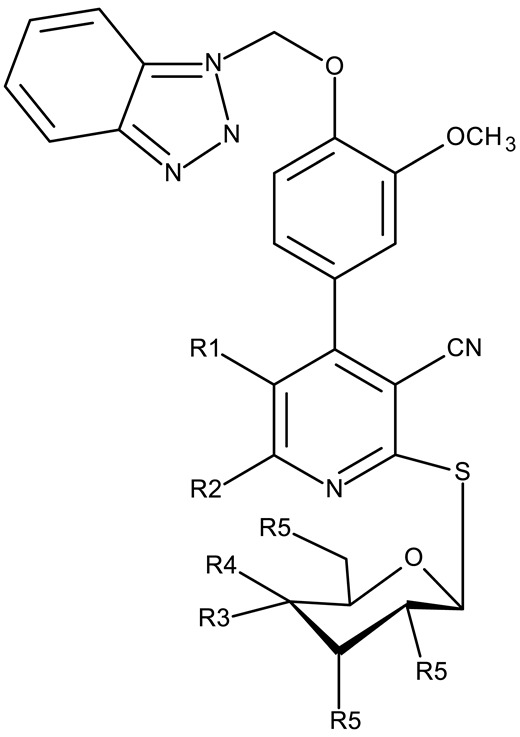	R1 = cyano, R2 = amino, R3 = R5 acetoxy, R4 = H (**ND 111**) R1 = acetyl, R2 = hydroxyl, R3 = R5 acetoxy, R4 = H (**ND 112**) R1 = cyano, R2 = hydroxyl R3 = R5 = acetoxy, R4 = H (**ND 113**) R1 = ethoxycarbonyl, R2 = hydroxyl R3 = R5 = acetoxy, R4 = H (**ND 114**) R1 = cyano, R2 = amino, R3 = H, R4 = R5 = acetoxy (**ND 115**) R1 = acetyl, R2 = hydroxyl R3 = H, R4 = R5 = acetoxy (**ND 116**) R1 = cyano, R2 = hydroxyl R3 = H, R4 = R5 = acetoxy (**ND 117**) R1 = ethoxycarbonyl, R2 = hydroxyl R3 = H, R4 = R5 = acetoxy (**ND 118**) R1 = cyano, R2 = amino, R3 = R5 = hydroxyl, R4 = H (**ND 118**) R1 = acetyl, R2 = R3 = R5 = hydroxyl, R4 = H (**ND 120**) R1 = cyano, R2 = R3 = R5 = hydroxyl, R4 = H (**ND 121**) R1 = ethoxycarbonyl, R2 = R3 = R5 = hydroxyl, R4 = H (**ND 122**) R1 = cyano, R2 = amino, R3 = H, R4 = R5 = hydroxyl (**ND 123**) R1 = acetyl, R2 = R4 = R5 = hydroxyl, R3 = H (**ND 124**) R1 = cyano, R2 = R4 = R5 = hydroxyl, R3 = H (**ND 125**) R1 = ethoxycarbonyl, R2 = R4 = R5 = hydroxyl, R3 = H (**ND 126**)	**ND 111–126** (4a–h, 5a–h)	MCF-7: 32–180 μM	↑apoptosis by ↑BAX, ↑p53 and ↓Bcl-2	[48]
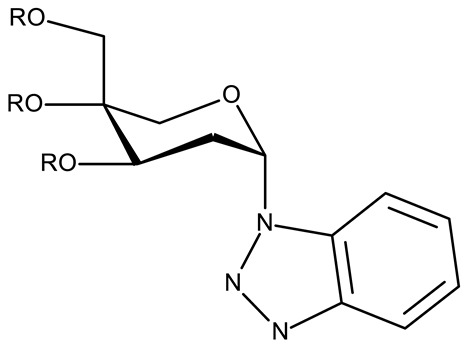	R = benzyl (**ND 127**) R = acetyl (**ND 128**)	**ND 127–128** (5, 6b)	HeLa: 2.9–9.94 μM	N/A	[49]
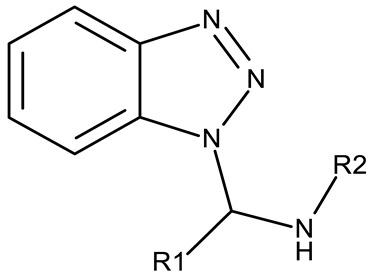	R1 = H, R2 = 3,5-dimethoxyphenyl (**ND 129**) R1 = H, R2 = 3,5-dinitrophenyl (**ND 130**) R1 = H, R2 = 4-aminopyridyl (**ND 131**) R1 = H, R2 = 3,5-dimethoxy-4-methylphenyl (**ND 132**) R1 = ethyl, R2 = 4-chlorophenyl (**ND 133**) R1 = methyl, R2 = 3-chlorophenyl (**ND 134**) R1 = 3-chlorophenyl, R2 = 4-nitrophenyl (**ND 135**) R1 = 4-chlorophenyl, R2 = 4-nitrophenyl (**ND 136**) R1 = 4-(N,N’-dimetzlamino = phenyl, R2 = 4-nitrophenyl (**ND 137**) R1 = 4-fluorophenyl, R2 = 4-nitrophenyl (**ND 138**) R1 = H, R2 = phenyl (**ND 139**) R1 = H, R2 = 4-nitrophenyl (**ND 140**) R1 = H, R2 = 4-chlorophenyl (**ND 141**) R1 = H, R2 = 4-metoxyphenyl (**ND 142**) R1 = H, R2 = 3-chlorophenyl (**ND 143**) R1 = ethyl, R2 = 3-chlorophenyl (**ND 144**)	**ND 129–144** (6a–p)	MCF-7: 0.012–18.5 μM A549: 0.18–22.9 μM Colo-205: 0.34–12.6 μM A2780: 0.07–13.5 μM	N/A	[50]
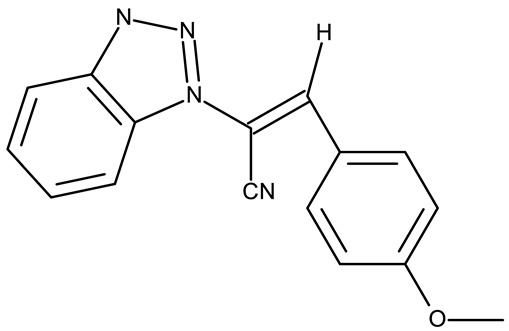	-	**ND 145** (34)	HeLa: 0.02 μM PC-3: 0.08 μM MCF-7: 0.1 μM SKMEL-28: 0.2 μM SKMES-1: 0.6 μM HepG1: 0.8 μM	↑apoptosis by ↑p53 signaling pathway; ↑cell cycle arrest (G2/M)	[51]
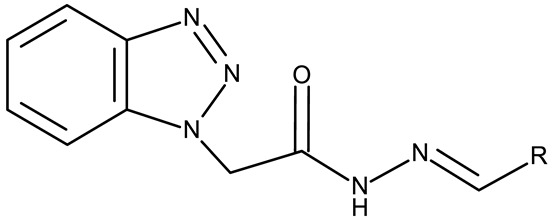	R = 2-hydroxyphenyl (**ND 146**) R = 2,3-dihydroxyphenyl (**ND 147**) R = 2,4-dihydroxyphenyl (**ND 148**) R = indol-3yl (**ND 149**)	**ND 146–149** (3d–f, 3q)	ND 146–149 OVCAR-3: 0.13–0.037 μM ND 147 HL-60: 0.025 μM)	↑apoptosis; ↑cell cycle arrest (G2/M); ↓caspase 3 activation	[52]
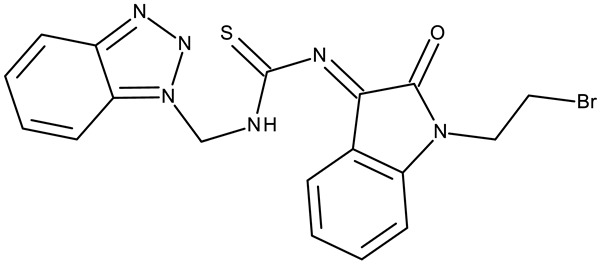	-	**ND 150** (5l)	MCF-7: 2.88 μM	↓COX; ↓PI3K	[53]
**Bis-benzotriazole hybrids**					
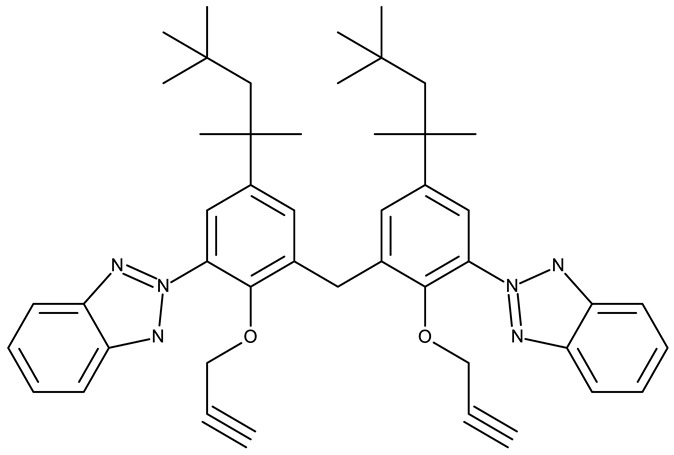	-	**ND 151** (TAJ1)	MCF-7: 4.04 μM HeLa: 6.08 μM	N/A	[54]
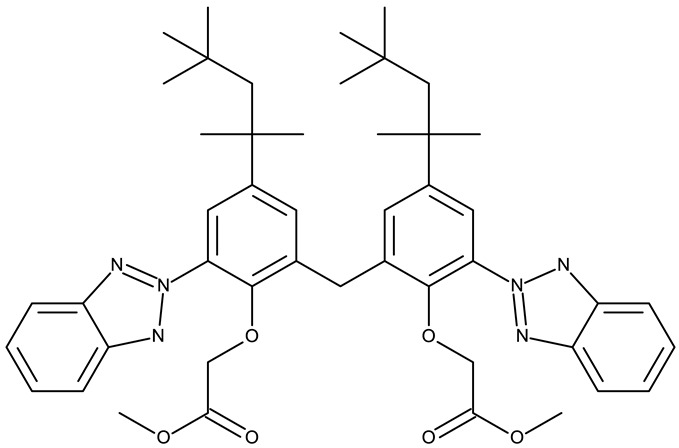	-	**ND 152** (TAJ4)	MCF-7: 3.18 ± 0.11 μM HeLa: 8.12 ± 0.43 μM	N/A	[55]
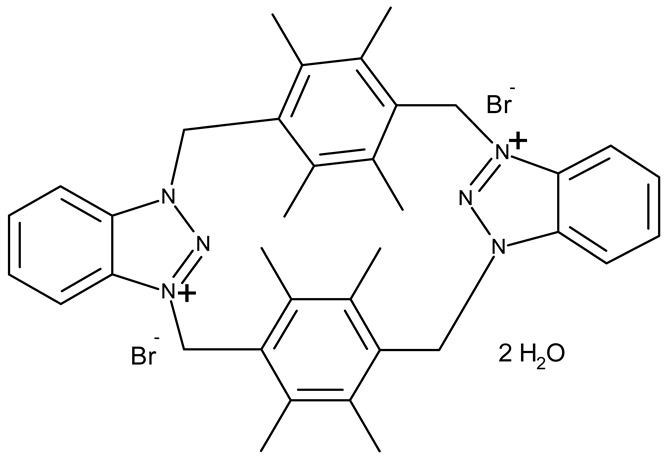	-	**ND 153** (BTD)	MCF-7: 83.45 μM	N/A	[56]

**Table 2:** C-substituted and fused derivatives of a benzotriazole (N/A—no data available within the article; stereochemistry is shown only when reported in the original studies; the original compound IDs are given in parentheses; ↑ indicates induction or up-regulation; and ↓ indicates inhibition or down-regulation).

Chemical Structure		Compound ID	IC_50_ (μM or μg/mL), Inhibition Rate (%)	Mechanism of Action	Reference
Core Structure	Substituents				
**C-substituted derivatives**					
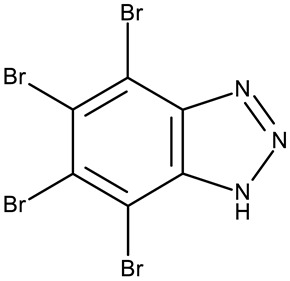	-	**CD 1** (TBB)	C4-2: 28–28.4 μM	↓CK-2 ↓NFκB p65	[67]
	-	**CD 1** (TBB)	WiDr: 0.15 μM	↓CK2; ↓XRCC1 phosphorylation	[68]
	-	**CD 1** (TBB)	N/A	↑apoptosis; ↓PTEN and Akt phosphorylation	[69]
	-	**CD 1** (2)	CCRF-CEM: 7.5–94.3 μM (24–48 h) MCF-7: 55.1–102.1 μM (24–48 h)	N/A	[70]
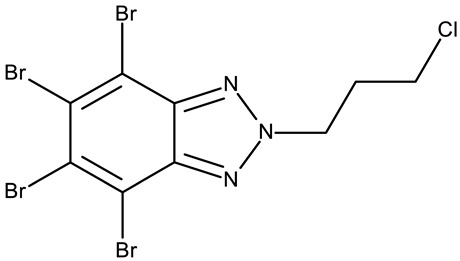	-	**CD 2** (4)	CCRF-CEM: 76.6–90.6 μM (24–48 h) MCF-7: 53.4–74.1 μM (24–48 h)	does not inhibit CK2	
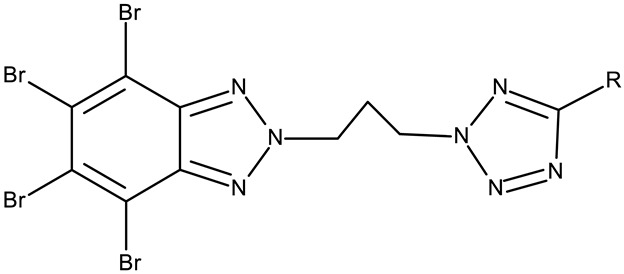	R = phenyl (**CD 3**) R = 4-methylphenyl (**CD 4**) R = 4-chlorophenyl (**CD 5**) R = 2-chlorophenyl (**CD 56**)	**CD 3–6** (8a–d)	CCRF-CEM: 12.9–94 μM (24–48 h) MCF-7: 1.3–80.3 μM (24–48 h)		
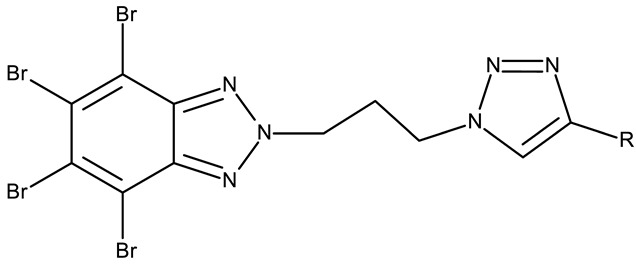	R = H (**CD 7**) R = hydroxymethyl (**CD 8**) R = ethyl ester (**CD 9**) R = carboxylic acid (**CD 10**) R = 2-carboxyethyl (**CD 11**)	**CD 7–11** (19–23)	MCF-7: 53–119% CCRF-CEM: 48–93%	N/A	[71]
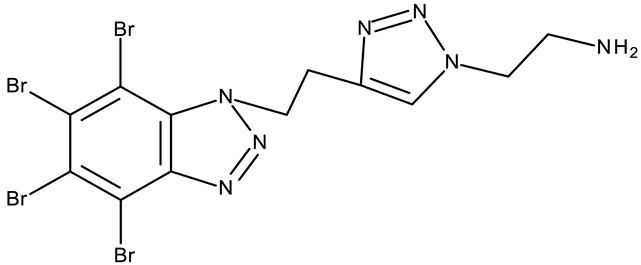	-	**CD 12** (10)	Jurkat T: 14.2 μM L1210: 20.7 μM MDA-MB-231: 17.4 μM MCF-7: 17 μM	↑apoptosis (intrinsic pathway)	[72]
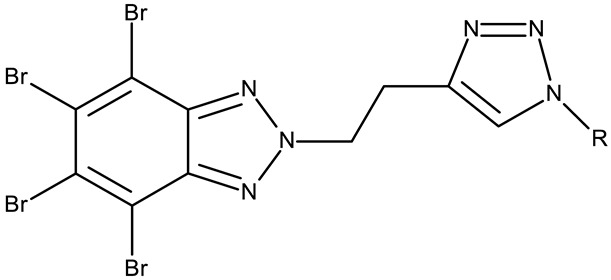	R = 4-aminobutyl (**CD 13**) R = 2-aminoethyl (**CD 14**)	**CD 13–14** (14,16)	CD 13 Jurkat T: 8.7 μM L1210: 11.42 μM MDA-MB-231: 9.7 μM MCF-7: 9.0 μM CD 14 Jurkat T: 7.9 μM L1210: 14.3 μM MDA-MB-231: 14 μM MCF-7: 11.6 μM		
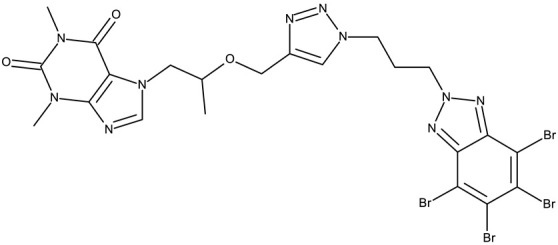	-	**CD 15** (42)	CCRF-CEM: 6.5 μM MCF-7: 80 μM	N/A	[73]
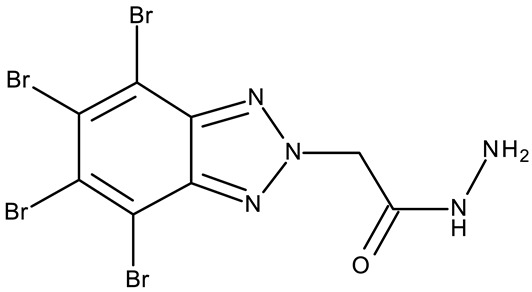	-	**CD 16** (5)	MCF-7: 9.1 μM A549: 6.3 μM	↑Bax; ↓CK-2	[74]
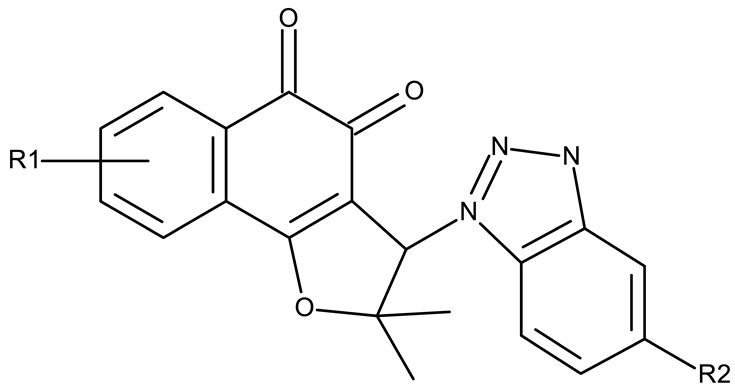	R1 = R2 = H (**CD 17**) R1 = 7-methyl, R2 = H (**CD 18**) R1 = 7-Br, R2 = H (**CD 19**) R1 = 9-methoxy, R2 = H (**CD 20**) R1 = H, R2 = *n*-butyl (**CD 21**)	**CD 17–21** (5a–b, 5f–g, 5k)	CD 17–20 MCF-7: 1.53–2.14 μM HepG2: 1.74–13.79 μM A549: 2.06–14.72 μM CD 21 MCF-7: 2.64 μM HepG2: 2.4 μM A549: 0.49 μM	↑apoptosis (intrinsic pathway) by ↑NQO1 and ↑ROS; ↑cell cycle arrest (G0/G1)	[75]
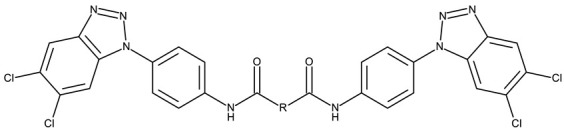	R = propane-1,3-diyl (**CD 22**) R = vinylene (**CD 23**)	**CD 22–23** (3b, 3d)	CCRF-CEM: 0.07–5.5 μM WIL-2NS: 23–>100 μM CCRF-SB: 0.35–8.5 μM SK-MEL28: 2.6–>100 μM SK-MES1: 6.8–>100 μM DU145: 5.1–>100 μM HeLa: 5.4–>100 μM	↑apoptosis	[76]
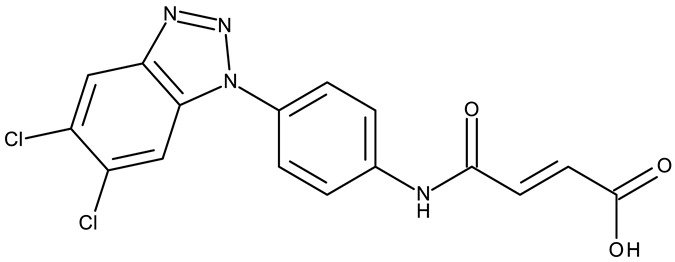	-	**CD 24** (4d)			
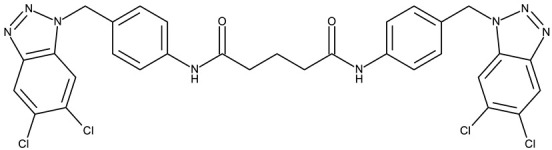	-	**CD 25** (9b)			
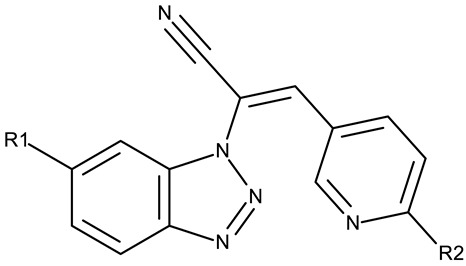	R1 = H, R2 = Cl (**CD 26**) R1 = H, R2 = methoxy (**CD 27**) R1 = Cl, R2 = Cl (**CD 28**) R1 = Cl, R2 = methoxy (**CD 29**)	**CD 26–35** (1.1–1.4, 2.1–2.6)	VX2: 3.08–56.55 μM A549: 5.47–59.41 μM MGC-803: 3.04–21.77 μM MKN45: 3.04–12.55 μM	N/A	[77]
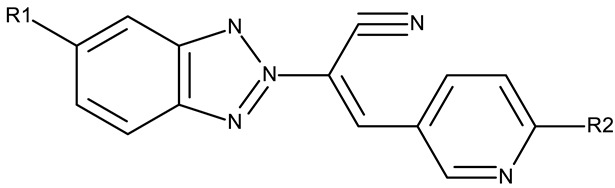	R1 = H, R2 = Cl (**CD 30**) R1 = H, R2 = methoxy (**CD 31**) R1 = Cl, R2 = Cl (**CD 32)** R1 = Cl, R2 = methoxy (**CD 33**)				
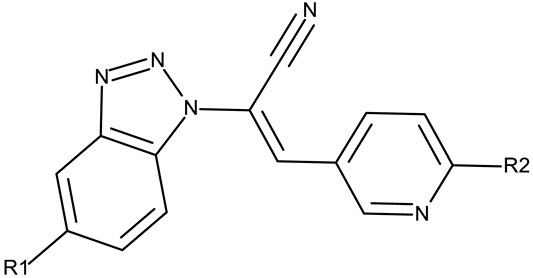	R1 = Cl, R2 = Cl (**CD 34**) R1 = Cl, R2 = methoxy (**CD 35**)				
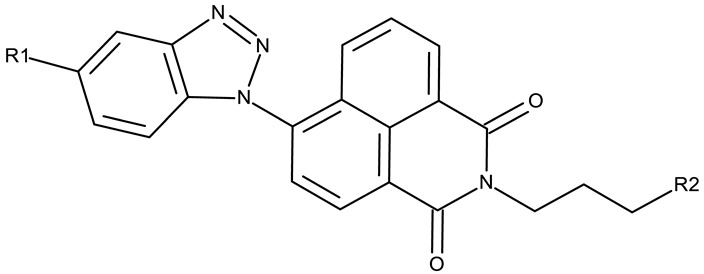	R1 = F, R2 = dimethylamino (**CD 36**) R1 = H, R2 = piperazin-1-yl (**CD 37**) R1 = Cl, R2 = piperazin-1-yl (**CD 38**)	**CD 36–38** (1b, 3a, 3c)	A549: 6.73–>20 μM SK-0V-3: 8.94–>20 μM HT-29: 8.34–>20 μM HL-60: 13.03–>60 μM PC-3: 10.85–>20 μM HepG2: 12.12–>20 μM MDA-MB-231: 8.85–>20 μM	↑apoptosis (intrinsic pathway) by ↓BCL2, ↑Bax, ↑Cyt C, ↑cleaved caspase-9/-3 and ↑cleaved PARP; ↑cell cycle arrest (G0/G1) ↑DNA damage; ↑autophagy by ↑LC3B-II, ↑Beclin1 and ↓p62	[78]
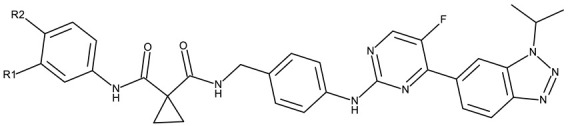	R1 = F, R2 = H (**CD 39**) R1 = H, R2 = F (**CD 40**) R1 = Cl, R2 = H (**CD 41**) R1 = H, R2 = Cl (**CD 42**) R1 = Br, R2 = H (**CD 43**) R1 = H, R2 = Br (**CD 44**) R1 = methoxy, R2 = H (**CD 45**) R1 = H, R2 = methoxy (**CD 46**) R1 = methyl, R2 = H (**CD 47**) R1 = H, R2 = methyl (**CD 48**) R1 = cyano, R2 = H (**CD 49**) R1 = H, R2 = cyano (**CD 50**) R1 = trifluoromethyl, R2 = H (**CD 51**) R1 = H, R2 = trifluoromethyl (**CD 52)** R1 = R2 = H (**CD 53**) R1 = trifluoromethyl, R2 = Cl (**CD 54**)	**CD 39–54** (12A–P)	**CD 39–54** SKOV-3: 0.029–0.915 μM SiHa: 0.009–0.231 μM **CD 53** HeLa: 0.024 Μm MCF-7: 0.01 μM 4T1: 0.068 μM OVCAR-5: 0.052 μM A549: 0.035 μM H460: 0.041 μM	↑apoptosis; ↑cell cycle arrest (G2/M); ↓CDKs and ↓FLTs activity	[79]
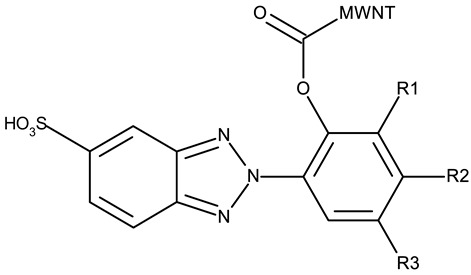	R1 = phenyl, R2 = phenyl, R3 = H (**CD 55**) R1 = nitroso, R2 = phenyl, R3 = phenyl (**CD 59**) R1 = H, R2 = H, R3 = nitro (**CD 57**) MWNT = multiwalled carbon nanotube	**CD 55–57** (B, C, D)	MKN-45: 0.002–0.06 μg/mL SW742: 0.001 μg/mL	N/A	[80]
**Fused derivatives**					
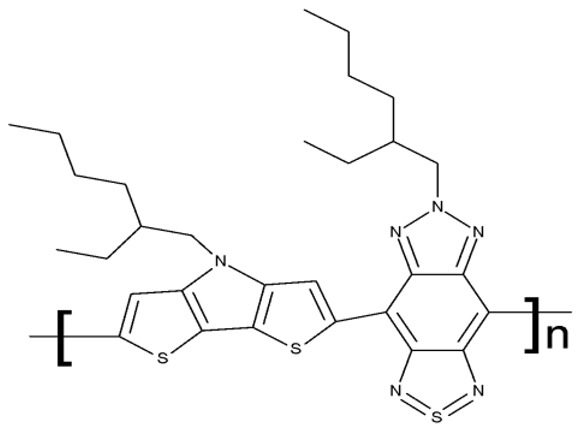	-	**FD 1** (from surface-modified NPs ± cRGD peptide)	NPs+ laser: C6: 9.67 μM U87 MG: 28.76 μM GL261: 17.71 μM cRGD NPs + laser: C6: 6.18 μM U87 MG: 21.13 μM GL261: 9.97 μM	N/A	[81]

**Table 3:** Organometallic compounds containing a benzotriazole (N/A—no data available within the article; stereochemistry is shown only when reported in the original studies; the original compound IDs are given in parentheses; ↑ indicates induction or up-regulation; and ↓ indicates inhibition or down-regulation).

Chemical Structure		Compound ID	IC_50_ (μM or μg/mL), Inhibition Rate (%)	Mechanism of Action	Reference
Core Structure	Substituents				
**Organometallic compounds**					
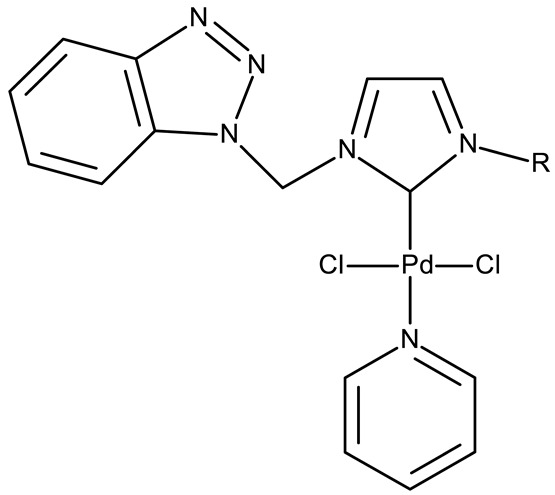	R = methyl (**OM 1**) R = butyl (**OM 2**)	**OM 1–4** (1a–d)	Caco-2: 162–376 μM MCF-7: 192–530 μM	N/A	[87]
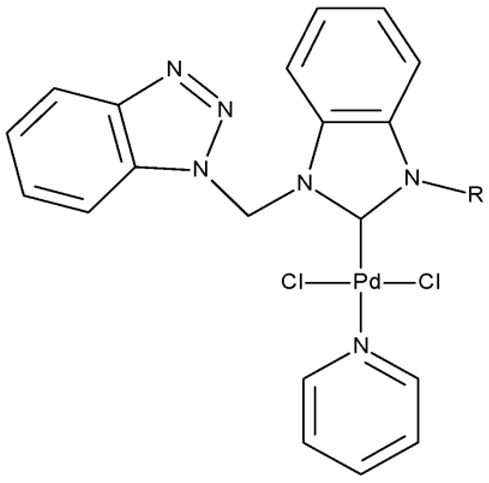	R = butyl (**OM 3**) R = benzyl (**OM 4**)				
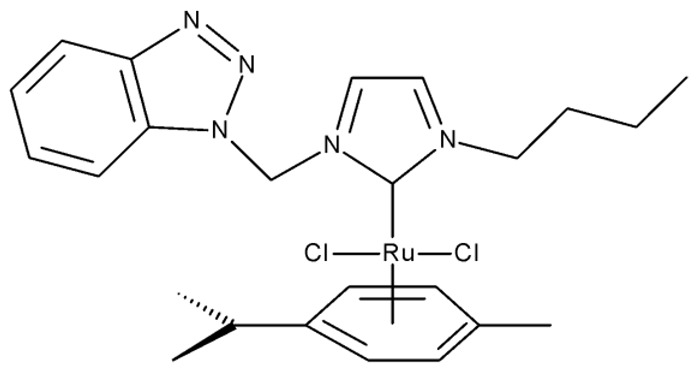	-	**OM 5** (3a)	Caco-2: 90–201 μM MCF-7: 137–407 μM		
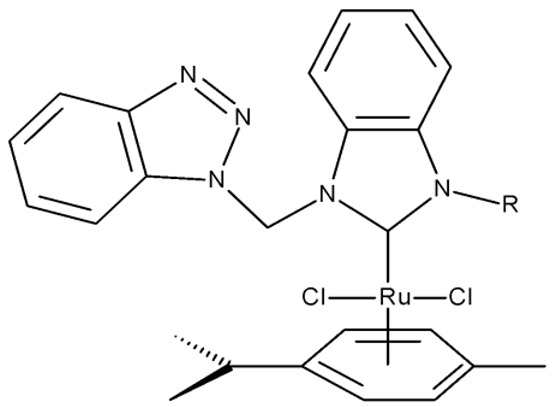	R = butyl (**OM 6**) R = benzyl (**OM 7**) R = 3,4,5-trimethoxybenzyl (**OM 8**)	**OM 6–8** (3b–d)			
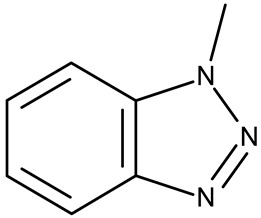 mebta	-	**OM 9** ([(CH3)2SnCl2(mebta)2])	MDA-MB-231: 20 μM	N/A	[88]
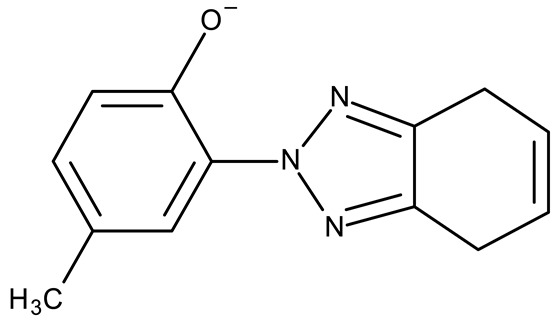 hmbt	-	**OM 10–18** ([Zn(hmbt)_2_(H_2_O)_2_]; [Zn(hmbt)(OAc)(H_2_O)_2_]; [Pd(bpy)(hmbt)]Cl; [Pt(bpy)(hmbt)]Cl [Pd(phen)(hmbt)]C; [Pt(phen)(hmbt)]Cl; [Ag_2_(hmbt)_2_]; [Ag(PPh_3_)(hmbt)]; [Rh(hmbt)_2_(H_2_O)_2_]Cl) (bpy- 2,2′-bipyridine, phen- 1,10-phenanthroline, PPh_3_+ triphenylphosphine)	**OM 17** MDA-MB231: 1.37 μM OVCAR-8: 1.75 μM **OM 14** MDA-MB231: 4.85 μM OVCAR-8: 2.99 μM **OM 15** MDA-MB-231: 5.24 μM OVCAR-8: 3.00 μM **OM 18** MDA-MB231: 7.52 μM OVCAR-8: 8.50 μM	N/A	[89]
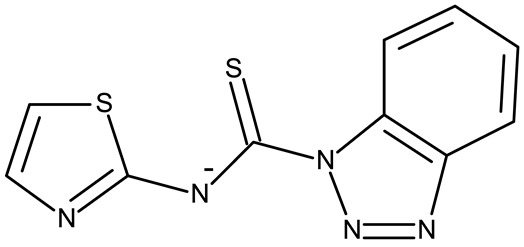 L	-	**OM 19–21** ([ZnL_2_]·4EtOH, [PdL(EtOH)_2_]·Cl, [PtL(EtOH)Cl])	MCF-7: 3.08–4.28 μg/mL	↓metastasis; ↓angiogenesis by ↓VEGF	[90]
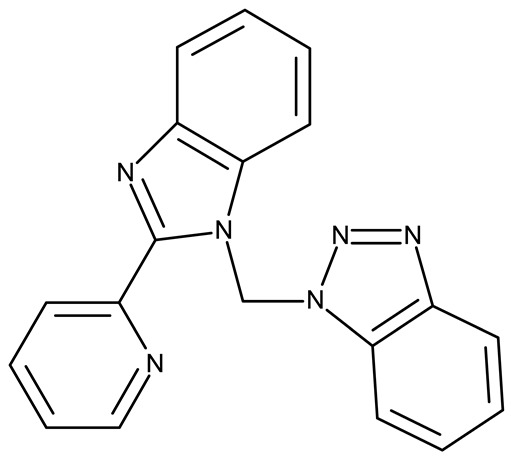 bmb	-	**OM 22** (Zn_2_(p-2-bmb)_2_(NO_3_)_4_)	MCF-7: 33.0 μM QBC939: 37.2 μM SHSY5Y: 30.3 μM EC109: 36.3 μM	↑apoptosis (intrinsic pathway); ↑cell cycle arrest (G0/G1)	[91]
	-	**OM 23–24** (Cu(p-2-bmb)(OH)(ClO_4_), **OM 3** (Co_2_(p-2-bmb)_2_Cl_4_)	OM 23 SMMC7721: 39.2 μM BGC823: >80 μM HCT116: 43.5 μM	OM 23: ↑apoptosis (intrinsic pathway); ↑cell cycle arrest (G2/M)	[92]
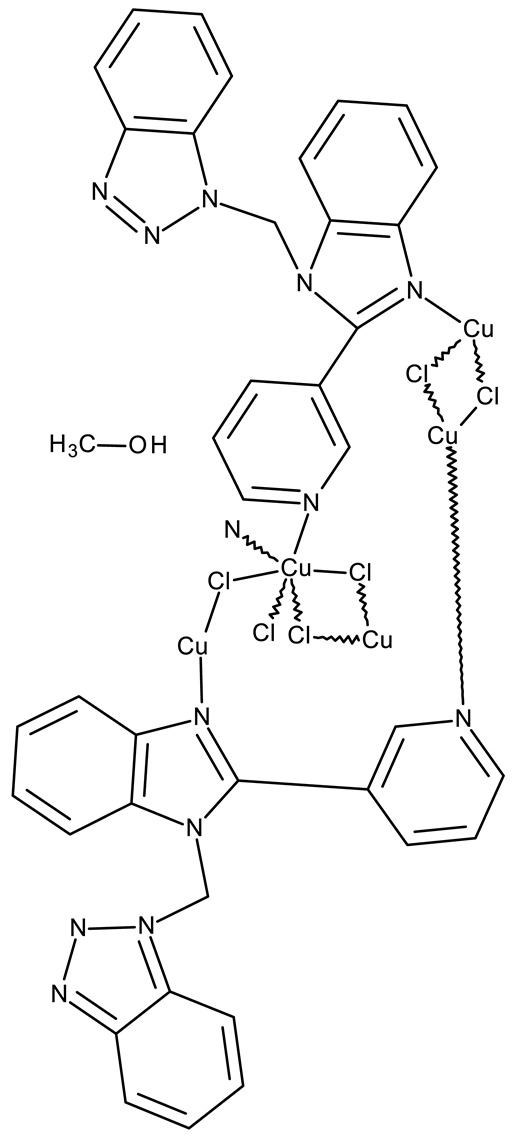	-	**OM 25** (Cu (II))	HeLa: 24.2–7.18 μM (24–72 h) SGC-7901: 27.64–8.35 μM (24–72 h)	↑apoptosis (intrinsic pathway) by ↑ROS; ↑cell cycle arrest (G1) by ↓ cyclinD1/cdk4 and ↓pRb/E2F1	[93]
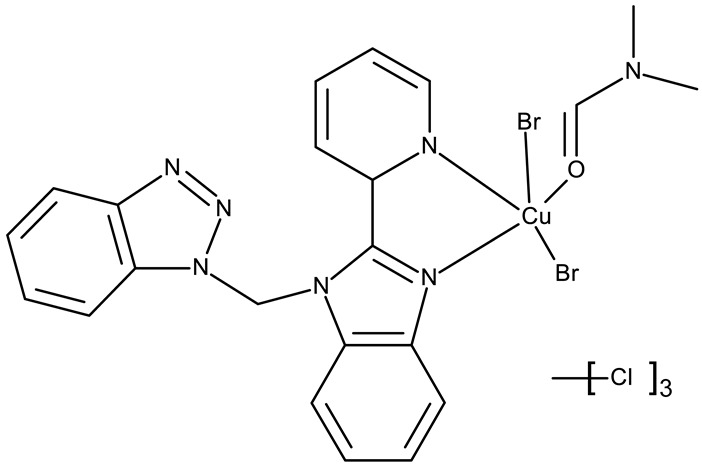	-	**OM 26** (3)	MCF-7: 35.5–54.8 μM EC109: 14.05–28.75 μM SHSY5Y: 34.37–59.78 μM QBC939: 31.71–40.4 μM	N/A	[94]
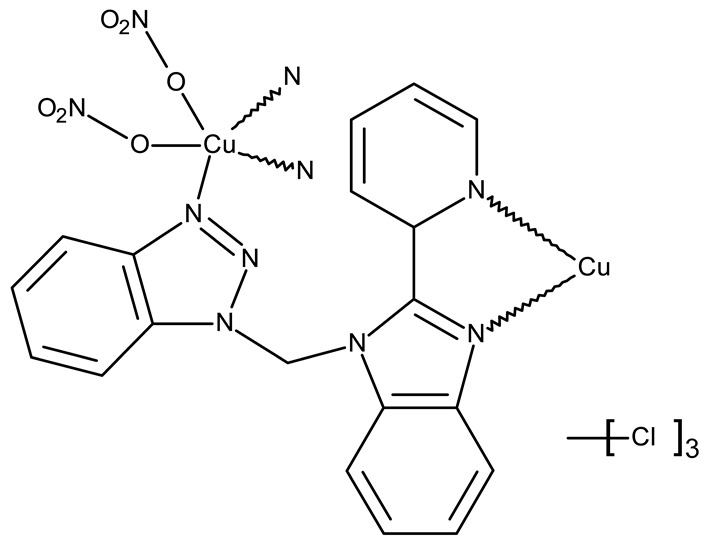		**OM 27** (4)			

**Table 4:** In vivo evidence of benzotriazole derivatives in cancer (N/A—no data available within the article; ↑ indicates induction or up-regulation; and ↓ indicates inhibition or down-regulation).

Compound ID	Cancer Model	Treatment	Control	Tumor Weight	Toxicity	Mechanism of Action	Reference
**CD 1**	22Rv1 cells (prostate cancer), orthotopic xenograft	i.v., 0.02 mg/kg on days 1, 4 and 7	TBG-RNAi-F7	reduced tumor weight	N/A	↓CK2; ↓AR; ↓NF-κB p65	[67]
**CD 1**	WiDr (colon cancer) xenografts, s.c.	i.p., 150 mg/kg, twice daily+ irradiation, 5 days	DMSO	delayed tumor growth	N/A	↓CK2	[68]
**CD 21**	HepG2 (hepatocellular) xenograft, s.c.	i.v., 20 mg/kg, every two days, 19 days	saline	tumor inhibition (52.3%)	none observed	N/A	[75]
**CD 53**	SiHa cells (squamous cell carcinoma), s.c.	p.o., 5–20 mg/kg, 30 days	cisplatin	tumor inhibition (51.25–79.29%)	none observed	N/A	[79]
**FD 1** (NPs)	GL261-luc cells (glioblastoma),injected in the right brain and C6 cells (glioma), orthotropic xenograft	NPs (1 mg/Kg) ± laser	PBS	marked tumor reduction (+ laser group)	no weight loss	↓CD31; ↓ki67 positive cells	[81]
**OM 25**	sarcoma murine cancer (S180), i.p.	i.p.; 1–10 mg/kg, 7 days	cisplatin, saline	tumor inhibition (42.29–59.86%)	no weight loss; the 10 mg/kg dose induced some hepatotoxicity	↑apoptosis; ↑cell cycle arrest (G1) by ↓cyclinD1/cdk4 pathway	[93]

## Data Availability

The data supporting the findings of this study are available within the article and its Supplementary Materials. Any further inquiries should be addressed to the corresponding author.

## Supplementary Materials

The following supporting information can be downloaded at https://www.mdpi.com/article/10.3390/ph19010077/s1. Table S1. Data extraction in vitro (N/A—no data available). Table S2. Data extraction in vivo (N/A—no data available). Table S3. QUIN tool-Risk of bias (Reviewer 1). Table S4. QUIN tool-Risk of bias (Reviewer 2). Table S5. QUIN tool-Risk of bias (Team discussions). Table S6. SYRCLE Risk of bias (Reviewer 1). Table S7. SYRCLE Risk of bias (Reviewer 2).
