# Supplementary material for: Benzotriazole in Cancer: A Systematic Review on Preclinical Evidence and Structure–Activity Relationship
Source: Pharmaceuticals (Basel). 2025 Dec 30;19(1):77. doi: 10.3390/ph19010077 (PMC12844763; doi:10.3390/ph19010077)
Supplement: Supplementary file 1 [file pharmaceuticals-19-00077-s001.zip › pharmaceuticals-4051096-supplementary.pdf]

Benzotriazole in Cancer: A Systematic Review on Preclinical Evidence and Structure–Activity Relationship

Authors: Gabriel Mardale [1,2], Alexandra Prodea [1,2,*], Andreea Munteanu [1,2], Mihaela Jorgovan [1,2], Sabina Mardale [3], Victor Cristian Dumitrascu [3] and Codruța Șoica [1,2]

Affiliation

[1] Faculty of Pharmacy, “Victor Babes” University of Medicine and Pharmacy, Eftimie Murgu Square, No. 2, 300041 Timișoara, Romania

[2] Research Center for Experimental Pharmacology and Drug Design (X-Pharm Design), “Victor Babes” University of Medicine and Pharmacy, Eftimie Murgu Square, No. 2, 300041 Timișoara, Romania

[3] Timiș County Emergency Clinical Hospital Pius Brinzeu, Liviu Rebreanu 156, 300723 Timișoara, Romania

* Correspondence: alexandra.ulici@umft.ro

Table S1. Data extraction *in vitro* (N/A- no data available).

Classification	First author's name and year of publication	Title	Compounds ID	Types of cancer	IC50 (µM or µg/ml), Inhibition rate (%)	Control	Mechanism of action	Reference
C-substituted	Fan 2020	Discovery of 12O-A Novel Oral Multi-Kinase Inhibitor for the Treatment of Solid Tumor	12A-P	breast cancer (MCF-7, 4T1), lung cancer (A549, H460), cervical cancer (HeLa, SiHa), ovarian cancer (OVCAR-5, SKOV3)	12A-P: SK-OV-3 (0.029-0.915 µM), SiHa (0.009-0.231 µM), 12O: HeLa (0.024 µM), MCF-7 (0.01 µM), 4T1 (0.068 µM), OVCAR-5 (0.052 µM), A549 (0.035 µM), H460 (0.041 µM)	cisplatin	induction of apoptosis, G2/M phase cell cycle arrest, inhibit CDKs and FLTs activity	[1]

C-substituted	Shehata 2010	Reconstitution of PTEN activity by CK2 inhibitors and interference with the PI3-K/Akt cascade counteract the antiapoptotic effect of human stromal cells in chronic lymphocytic leukemia	TBB	leukemia (peripheral blood mononuclear cells from patients with CLL)	N/A	N/A	decreased the phosphorylation of PTEN and Akt, induction of apoptosis	[2]
C-substituted	Trembley 2019	CK2 pro-survival role in prostate cancer is mediated via maintenance and promotion of androgen receptor and NFκB p65 expression	TBB	prostate cancer (LNCaP, C4-2 and 22Rv1)	C4-2 (28-28.4 μM)	N/A	reduced CK-2 and NFκB p65 protein levels	[3]
C-substituted	Zwicker 2021	In vivo evaluation of combined CK2 inhibition and Irradiation in Human WiDr Tumours	TBB	colon cancer (WiDr)	WiDr (0.15 μM)	DMSO, DMSO+ irradiation	inhibits CK2, reduces XRCC1 phosphorylation	[4]
C-substituted	Chojnacki 2017	Synthesis, in vitro antiproliferative activity and kinase profile of new benzimidazole and benzotriazole derivatives	19-23	breast cancer (MCF-7), leukemia (CCRF-CEM)	19-23: MCF-7 (53-119%), CCRF-CEM (48-93%)	DMSO	N/A	[5]

C-substituted	El-Kardocy 2020	CK2 inhibition, lipophilicity and anticancer activity of new: N 1versus N 2-substituted tetrabromobenzotriazole regioisomers	5	breast cancer (MCF-7), lung cancer (A549)	5: MCF-7 (9.1 µM), A549 (6.3 µM)	doxorubicin	upregulation of Bax, inhibition of CK-2	[6]
C-substituted	Chojnacka 2016	Synthesis of polybrominated benzimidazole and benzotriazole derivatives containing a tetrazole ring and their cytotoxic activity	2,4, 8a-d	breast cancer (MCF-7), leukemia (CCRF-CEM)	2: CCRF-CEM to 24h (25.4 - 94.3 µM), MCF-7 to 24h (77.9 - 102.1 µM); 4: CCRF-CEM to 24h (81.6 - 90.6 µM), MCF-7 to 24h (59.2 - 74.1 µM); 8a-d: CCRF-CEM to 24h (31 to 8c - 94 µM), MCF-7 to 24h (5.1 to 8c - 80.3 µM); 2: CCRF-CEM to 48h (7.5 - 78.9 µM), MCF-7 to 48h (51.1 - 93.9 µM); 4: CCRF-CEM to 48h (76.6 - 82.1 µM), MCF-7 to 48h (53.4 - 60.3 µM); 8a-d: CCRF-CEM to 48h (12.9 to 8c - 94 µM), MCF-7 to 48h (1.3 to 8c - 64.8 µM)	doxorubicin, DMSO	does not inhibit CK2	[7]
C-substituted	Entezari 2013	Modification of carboxylated multiwall nanotubes with benzotriazole derivatives and study of their anticancer	B,C,D	gastric cancer (MKN-45), colon cancer (SW742)	B,C,D: MKN-45 (0.002-0.06 µg/mL), SW742 (0.001 µg/mL)	cisplatin, doxorubicin	N/A	[8]

		activities						
C-substituted	Swider 2015	Synthesis, biological activity and structural study of new benzotriazole-based protein kinase CK2 inhibitors	10,14,16	breast cancer (MDA-MB 231, MCF-7), human leukemia (Jurkat T), murine leukemia (L1210)	10: Jurkat T (14.2 μM), L1210 (20.7 μM), MDA-MB-231 (17.4 μM), MCF-7 (17 μM); 14: Jurkat T (8.7 μM), L1210 (11.42 μM), MDA-MB-231 (9.7 μM), MCF-7 (9.0 μM); 16: Jurkat T (7.9 μM), L1210 (14.3 μM), MDA-MB-231 (14 μM), MCF-7 (11.6 μM)	doxorubicin	induces apoptosis by inhibiting CK2	[9]
C-substituted	Ibba 2021	Synthesis, Antitumor and Antiviral In Vitro Activities of New Benzotriazole-Dicarboxamide Derivatives	3b, 3d, 4d,9b	leukemia (CCRF-CEM, WIL-2NS, CCRF-SB), melanoma (SK-MEL28), lung cancer (SK-MES1), prostate cancer (DU145), cervical cancer (HeLa)	3b, 3d, 4d, 9b: CCRF-CEM (0.07-5.5 μM), WIL-2N (23- >100 μM), CCRF-SB (0.35-8.5 μM), SK-MEL28 (2.6- >100 μM), SK-MES1 (6.8- >100 μM), DU145 (5.1- >100 μM), HeLa (5.4- >100 μM)	doxorubicin, vincristine	3b induced apoptosis after 96 hours	[10]

C-substituted	Wu 2021	Design, synthesis, and biological evaluation of 3-(1-benzotriazole)-nor-β-lapachones as NQO1-directed antitumor agents	5a-b, 5f-g, 5k	breast cancer (MCF-7), lung cancer (A549), hepatocellular carcinoma (HepG2)	5a-b: MCF-7 (1.99 -2.14 μM), HepG2 (1.74-1.92 μM), A549 (5.14-10.7 μM); 5f-g: MCF-7 (1.53 -1.75 μM), HepG2: (2.03-13.79 μM), A549: (2.06-14.72μM), 5k: MCF-7 (2.64 μM), HepG2 (2.4 μM), A549 (0.49 μM)	Nor-β-lapachone	5k induced cell apoptosis through activation of NQO1 → redox cycle → ROS accμMulation; mitochondrial membrane depolarization and arrested tμMor cell in G0/G1 phase	[11]
C-substituted	Borowiecki 2018	Synthesis of novel proxyphylline derivatives with dual Anti-Candida albicans and anticancer activity	42	breast cancer (MCF-7), leukemia (CCRF-CEM)	42: CCRF-CEM (6.5 μM), MCF-7 (80 μM)	N/A	N/A	[12]
C-substituted	Li 2019	Synthesis and anticancer activity of benzotriazole derivatives	1.1-1.4, 2.1-2.6	squamous cell carcinoma (VX2), lung cancer (A549), gastric cancer (MGC-803, MKN45)	1.1-1.4: VX2 (5.36-56.55 μM), A549 (10.52-59.41 μM), MGC-803 (4.65-21.77 μM), MKN45 (5.75-11.77 μM); 2.1-2.6: VX2 (3.8-39.94 μM), A549 (5.47-45.91 μM), MGC-803 (4.59-18.72 μM), MKN45 (3.04-12.55 μM)	gefitinib	N/A	[13]

C-substituted	Wang 2022	Design, synthesis and bioactivity of novel naphthalimide-benzotriazole conjugates against A549 cells via targeting BCL2 G-quadruplex and inducing autophagy	1b, 3a, 3c	lung cancer (A549), ovarian cancer (SK-OV-3), colon cancer (HT-29), prostate cancer (PC-3), promyelocytic leukemia (HL-60), breast cancer (MDA-MB-231), hepatocellular carcinoma (HepG2)	3a: A549 (6.73 μM), SK-0V-3 (8.94 μM), HT-29 (8.34 μM), HL-60 (13.03 μM), PC-3 (10.85 μM), HepG2 (14.00 μM), MDA-MB-231 (8.85 μM); 1b: A549 (19.35 μM), SK-0V-3 (13.14 μM), HT-29 (10.21 μM), HL-60, PC-3, HepG2, MDA-MB-231 (>20 μM); 3c: A549, SK-0V-3, HT-29, HL-60, PC-3 (>20 μM), HepG2 (12.12 μM), MDA-MB-231 (9.53 μM)	cisplatin	3a: induces mitochondrial apoptosis via the intrinsic pathway by (↓BCL2, ↑Bax, Cyt C release, ↑cleaved caspase-9/-3, cleaved PARP); induces cell cycle arrest G0/G1 phase; induces DNA damage; induces autophagy (↑LC3B-II, ↑Beclin1, ↓p62)	[14]
Fused derivative	He 2022	NIR-II absorptive dithienopyrrole-thiadiazolobenzotriazole conjugated polymer for photoacoustic imaging-guided glioblastoma multiforme photothermal therapy	PT NPs, cRGD@PT NPs	glioblastoma (U87 MG, C6, GL261), cervical cancer (HeLa)	PT NPs + laser: C6 (9.67 μM), U87 MG (28.76 μM), GL261 (17.71 μM); cRGD@PT NPs + laser: C6 (6.18 μM), U87 MG (21.13 μM), GL261 (9.97 μM)	N/A	N/A	[15]
N-substituted	Alraqa 2020	Design, click conventional and microwave syntheses, DNA binding, docking and anticancer studies of	4a-e, 6a-e, 8a-j	lung cancer (A549, H1299)	4a-e: A549 (78-84%), H-1229 (70-90%); 6a-e: A549 (78-89%), H-1229 (78-90%); 8a-j: A549 (81-89%), H-1229 (75-92%)	DMSO	N/A	[16]

		benzotriazole-1,2,3-triazole molecular hybrids with different pharmacophores						
N-substituted	Anusha 2022	SYNTHESIS AND BIOLOGICAL EVALUATION OF SUBSTITUTED MANNICH BASES OF BENZOTRIAZOLE DERIVATIVES AS ANTICANCER AGENTS	6a-p	breast cancer (MCF-7), lung cancer (A549), colon cancer (Colo), ovarian cancer (A2780)	6A-P: MCF-7 (0.012-18.5 μM), A549 (0.18-22.9 μM), Colo-205 (0.34-12.6 μM), A2780 (0.07-13.5 μM)	etoposide	N/A	[17]
N-substituted	Aziz 2024	Identification of dimethyl 2,2'-((methylenebis(2-(2H-benzo[d][1,2,3]triazol-2-yl)-4-(2,4,4-trimethylpentan-2-yl)-6,1phenylene))bis(oxy))diacetate (TAJ4) as antagonist of NEK-Family: a future for potential drug discovery	TAJ4	breast cancer (MCF-7), cervical cancer (HeLa)	TAJ4: HeLa (8.12 μM), MCF-7 (3.18 μM)	cisplatin, doxorubicin	N/A	[18]
N-substituted	Fabitha 2024	Novel fused pyran derivatives induce apoptosis and target cell cycle progression in anticancer efficacy	6e, 8e, 10e, 12e, 14e	breast cancer (MCF-7), lung cancer (A549), colon cancer (HCT 116)	6e: MCF-7 (12.47 μM), 8e: HCT116 (21.43 μM)	cisplatin	arrest the cell cycle in G0/G1, S and G2/M phases	[19]

		against multiple cell lines						
N-substituted	Garton 2021	Synthesis and antiproliferative evaluation of 2-deoxy-n-glycosylbenzotriazoles/imidazoles	5, 6b	breast cancer (MDA-MB-231), lung cancer (A549, HCC87), cervical cancer (HeLa)	5: HeLa (2.9 μM); 6b: HeLa (9.94 μM)	DMSO	N/A	[20]
N-substituted	Kassab 2018	Novel benzotriazole N-acylarylhydrazone hybrids: Design, synthesis, anticancer activity, effects on cell cycle profile, caspase-3 mediated apoptosis and FAK inhibition	3d-f, 3q	leukemia(HL-60), ovarian cancer (OVCAR-3)	3d: OVCAR-3 (0.13 μM); 3e: HL-60 (0.025 μM), OVCAR-3 (0.029 μM); 3f: OVCAR-3 (0.037 μM); 3q: OVCAR-3 (0.028 μM)	doxorubicin	3e inhibits the activation of caspase 3, induces apoptosis, arrests cell cycle in G2/M phase in OVCAR-3 and HL-60	[21]
N-substituted	Khayyat 2021	Design, synthesis, and antipoliferative activities of novel substituted imidazole-thione linked benzotriazole derivatives	BI1-12	breast cancer (MCF-7), leukemia (HL-60), colon cancer (HCT-116)	BI1-12: MCF-7 (2.29-38.2 μM), HL-60 (0.4-37.1 μM), HCT-116 (1.51-17.5 μM)	DMSO	BI9 induced G2/M cycle arrest, apoptosis through PARP cleavage and regulated the expression of BAX and Bcl-2 proteins	[22]

N-substituted	Khodair 2019	Discovery of New S-Glycosides and N-Glycosides of Pyridine-biphenyl System with Antiviral Activity and Induction of Apoptosis in MCF7 Cells	4a-h, 5a-h	breast cancer (MCF-7)	4a-h: MCF-7 (32-100 μM) ; 5a-h: MCF-7 (32-180 μM)	cisplatin	4f, 4g, 5f, 5h induced apoptosis, increased the apoptotic gene BAX and p53 and decreased anti-apoptotic gene Bcl-2	[23]
N-substituted	Korcz 2018	Synthesis, structure, chemical stability, and in vitro cytotoxic properties of novel quinoline-3-carbaldehyde hydrazones bearing a 1,2,4-triazole or benzotriazole moiety	5a-e, 7a-h, 9a-h	pancreatic cancer (DAN-G), lung cancer (LCLC-103H), cervical cancer (SISO)	5a-e: DAN-G (1.35-6.38 μM), LCLC-103H (1.23-6.29 μM), SISO (1.49-6.23 μM); 7a-h: DAN-G (2.56-6.37 μM), LCLC-103H (2.67- >20 μM), SISO (2.29-5.22 μM); 9a-h: DAN-G (2.48-6.59 μM), LCLC-103H (3.55- >20 μM), SISO (2.93-6.44 μM)	DMSO	N/A	[24]
N-substituted	Kumar 2020	p-TSA.H2O mediated one-pot, multi-component synthesis of isatin derived imidazoles as dual-purpose drugs against inflammation and cancer	5l	breast cancer (MCF-7)	5l: MCF-7 (2.88 μM)	doxorubicin	COX and PI3K mediated inhibition	[25]

N-substituted	Kuran 2020	Ester derivatives of salinomycin efficiently eliminate breast cancer cells via ER-stress-induced apoptosis	7	breast cancer (MCF-7, MDA-MB-231)	7: MCF-7 (4.1 μM), MDA-MB-231 (2.6 μM)	N/A	induced mitochondrial dependent apoptosis, induced G2/M cell cycle arrest, increased p53, increase the level of p-eIF2α (Ser51) and IRE1α proteins; increased γH2AX protein and modified guanine (8-oxoG)	[26]
N-substituted	Matheswari 2024	Synthesis, structural, multitargeted molecular docking analysis of anti-cancer, anti-tubercular, DNA interactions of benzotriazole based macrocyclic ligand	BTD	breast cancer (MCF-7)	BTD: MCF-7 (83.45 μM)	doxorubicin	N/A	[27]
N-substituted	Mermer 2022	Benzotriazole-oxadiazole hybrid Compounds: Synthesis, anticancer Activity, molecular docking and ADME profiling studies	4a-m	pancreatic cancer (PANC-1)	4a-m: PANC-1 (87.82 - 4650 μg/ml)	triton X, unexposed cells	N/A	[28]

N-substituted	Mioc 2022	Novel Triterpenic Acid-Benzotriazole Esters Act as Pro-Apoptotic Antimelanoma Agents	1-3	lung cancer (A549), cervical cancer (HeLa S3)	1: A375 25 μM (18.75%), 50 μM (30.2%); 2: A375 25 μM (12.6%), 50 μM (37.5); 3: A375 50 μM (23%)	betulinic acid, oleanolic acid, ursolic aid	induces mitochondrial apoptosis intrinsically by inhibiting cellular respiration	[29]
N-substituted	Pogaku 2019	The design and green synthesis of novel benzotriazoloquinolinyl spirooxindolopyrrolizidines: Antimycobacterial and antiproliferative studies	4a-p	lung cancer (A549), cervical cancer (HeLa S3)	4c: A549 (9.81 μM), HeLa S3 (20.4 μM); 4f: A549 (5.7 μM), HeLa S3 (11.6 μM); 4a-b: A549 (65.90 - >100 μM), HeLa S3 (>100 μM); 4d-e: A549 (61.7 - 22.4 μM), HeLa S3 (>100 - 47.4 μM); 4g-p: A549 (22.9- >100 μM), HeLa S3 (>100 μM)	cisplatin	N/A	[30]
N-substituted	Qadri 2023	Synthesis, biological evaluation and in silico investigations of benzotriazole derivatives as potential inhibitors of NIMA related kinase	TAJ1	breast cancer (MCF-7), cervical cancer (HeLa)	TAJ1: MCF-7 (4.04 μM), HeLa (6.08 μM)	displatin, doxorubicin	N/A	[31]
N-substituted	Zhang 2013	Synthesis, biological evaluation, and molecular docking studies of novel 1,3,4-oxadiazole derivatives possessing benzotriazole moiety	4-22	breast cancer (MCF-7), colorectal cancer (HT-29)	4-22: MCF-7 (5.68 - 45.16 μM); HT-29 (10.21 - 42.30 μM)	cisplatin	inhibits FAK and induces dose-dependent apoptosis in MCF-7	[32]

		as FAK inhibitors with anticancer activity						
N-substituted	Zoroddu 2024	Identification of 3-Aryl-1-benzotriazole-1-yl-acrylonitrile as a Microtubule-Targeting Agent (MTA) in Solid Tumors	34	breast cancer (MCF-7), cervical cancer (HeLa), prostate cancer (PC3), human melanoma (SKMEL-28), lung cancer (SKMES-1), liver cancer (HepG1)	34: HeLa (0.02 μM), PC-3 (0.08 μM), MCF-7 (0.1 μM), SKMEL-28 (0.2 μM), SKMES-1 (0.6 μM), HepG1 (0.8 μM)	DMSO, paclitaxel	induces cell cycle arrest in the G2/M phase, microtubule-stabilizing agent (MTA) that accelerates tubulin polymerization, induction of apoptosis by activating the p53 signaling pathway	[33]
Organometallic compound	El-Asmy 2013	Zinc(II), rutheniμM(II), rhodiμM(III), palladium(II), silver(I), platinum(II) and MoO2²⁺ complexes of 2-(2′-hydroxy-5′-methylphenyl)-benzotriazole as simple or primary ligand and 2,2′-bipyridyl, 9,10-	[Zn(hmbt)2(H2O)2], [Zn(hmbt)(OAc)(H2O)2], [Pd(bpy)(hmbt)]Cl, [Pt(bpy)(hmbt)]Cl, [Pd(phen)(hmbt)]Cl, [Pt(phen)(hmbt)]Cl, [Ag2(hmbt)2], [Ag(PPh3)(hm	breast cancer (MDA-MB-231), ovarian cancer (OVCAR-8)	[Ag(PPh3)(hmbt)]: MDA-MB-231 (1.37 μM), OVCAR-8 (1.75 μM) ; [Pd(phen)(hmbt)]Cl: MDA-MB-231 (4.85 μM), OVCAR-8 (2.99 μM) ; [Pt(phen)(hmbt)]Cl: MDA-MB-231 (5.24 μM), OVCAR-8 (3.00 μM) ; [Rh(hmbt)2(H2O)2]Cl: MDA-MB-231 (7.52	cisplatin	N/A	[34]

		phenanthroline or triphenylphosphine as secondary ligands: Structure and anticancer activity	bt)], [Rh(hmbt)2(H 2O)2]Cl		μM), OVCAR-8 (8.50 μM)			
Organometallic compound	Hu 2017	In vitro antitumor activity of novel benzimidazole-based Cu(II) complexes	Cu2 (p-2-bmp)2Br4 (1); Cu2(p-2-bmp)2Cl4 (2)	breast cancer (MCF7) esophagian cancer (EC109), neuroblastoma (SH-SY5Y), biliary duct cancer (QBC939)	3-4: MCF-7 (35.5 - 54.8 μM), EC109: (14.05 - 28.75 μM), SHSY5Y: (34.37 - 59.78 μM), QBC939: (31.71 - 40.4 μM)	cisplatin, DMSO	N/A	[35]

Organometallic compound	Li 2013	A new synthetic Cu(II) compound, [Cu3(p-3-bmb) 2Cl4·(CH3OH)2]n, inhibits tumor growth in vivo and in vitro	Cu (II)	cervical cancer (HeLa) and gastric cancer (SGC-7901)	Cu (II): HeLa 24h (24.2 μM), HeLa 48h (15.12 μM), HeLa 72h (7.18 μM); Cu (II): SGC-7901 24h (27.64 μM), SGC-7901 48h (16.17 μM), SGC-7901 72h (8.35 μM)	cisplatin	induces cell cycle arrest in the G1 phase (by regulating p53 and p21, ↓ cyclinD1/cdk4 and pRb/E2F1), induces apoptosis (via the mitochondrial pathway through the release of Cytochrome-C, activation of caspases 3 and 9, and PARP cleavage), stimulates the increase of reactive oxygen species that produce oxidative damage leading to apoptosis in HeLa cells and SGC-7901 cells	[36]
Organometallic compound	Mansour 2014	Complexes of N-(2-thiazolyl)-1H-benzotriazole-1-carbothioamide with Pd(II), Pt(II), and	[ZnL2]·4EtOH (1), [PdL(EtOH)2]·Cl (2) and [PtL(EtOH)Cl]	breast cancer (MCF-7)	1: MCF-7 (4.28 μg/ml); 2: MCF-7 (3.08 μg/ml); 3: MCF-7 (3.50 μg/ml)	cisplatin	reduced VEGF mRNA expression and prevent angiogenesis or	[37]

		Zn(II): Spectral, DFT, cytotoxicity and anti-angiogenic effect on MCF-7 cell line	(3)				metastasis of MCF7 cells	
Organometallic compound	Onar 2019	Palladium(II) and ruthenium(II) complexes of benzotriazole functionalized N-heterocyclic carbenes: Cytotoxicity, antimicrobial, and DNA interaction studies	1a-d, 3a-d	breast cancer (MCF-7), colon cancer (Caco-2)	1a-d: Caco-2 (162 - 376 μM), MCF-7 (192 - 530 μM); 3a-d: Caco-2 (90 - 201 μM), MCF-7 (137 - 407 μM)	cisplatin	N/A	[38]
Organometallic compound	Stamou 2024	Antiproliferative Activity of an Organometallic Sn(IV) Coordination Compound Based on 1-Methylbenzotriazole against Human Cancer Cell Lines	[(CH3)2SnCl2(mebta)2] (1)	breast cancer (MDA-MB-231)	1: MDA-MB-231 (20 μM)	N/A	N/A	[39]
Organometallic compound	Zhao 2015	Potential anticancer activity of benzimidazole-based mono/dinuclear Zn(II) complexes towards human carcinoma cells	Zn2(p-2-bmb)2(NO3)4 (1)	breast cancer (MCF-7), eshophagian cancer (EC109), neuroblastoma (SHSY5Y), cholangio tumor (QBC939)	1: MCF-7 72h (33.0 μM), QBC939 72h (37.2 μM), SHSY5Y 72h (30.3 μM), EC109 72h (36.3 μM)	cisplatin	1: activates apoptosis via the mitochondrial pathway, induces arrest in the cell cycle G0/G1 on the cell line SHSY5Y	[40]

Organometallic compound	Zhao 2017	Synthesis, chemical nuclease activity, and in vitro cytotoxicity of benzimidazole-based Cu(II)/Co(II) complexes	Cu(p-2-bmb)(OH)(ClO4) (1) and Co2(p-2-bmb)2Cl4 (2)	liver cancer (SMMC7721), gastric cancer (BGC823), colorectal cancer (HCT116)	1: SMMC7721 (39.2 µM), BGC823 (> 80 µM), HCT116 (43.5 µM)	cisplatin	1: activates apoptosis via the mitochondrial pathway , induces arrest in the cell cycle (G2/M) on the cell line SMMC7721; 2: N/A	[41]

Table S2. Data extraction *in vivo* (N/A- no data available).

Type of benzotriazole derivatives	First author's name and year of publication	Title	Compounds ID	Types of cancer and inoculation method	Animal model (type, age, sex)	Treatment (administration route, dose, duration)	Control	Tumor weight	Toxicity	Mechanism of action	Reference
C-substituted	Fan 2020	Discovery of 12O-A Novel Oral Multi-Kinase Inhibitor for the Treatment of Solid Tumor	12O	SiHa cells, s.c.	female BALB/c nude mice, 6-8 weeks	p.o. (gavage), 5, 10, or 20 mg/kg/day 12O for 30 days	cisplatin	Tumor growth inhibitions: 5 mg/kg (51.25%), 10 mg/kg (65.52%) and 20 mg/kg (79.29%)	no toxicity observed	N/A	[1]

C-substituted	Trembley 2019	CK2 pro-survival role in prostate cancer is mediated via maintenance and promotion of androgen receptor and NFκB p65 expression	TBB	22Rv1 cells, orthotopic xenograft	male NOD SCID gamma mice	i.v., 0.02mg/kg on days 1, 4 and 7	TBG-RNAi-F7	decreased tumor weight	N/A	loss of CK2 subunits as well as reduced androgen receptor and NFκB p65 total protein levels	[3]
C-substituted	Zwicker 2021	In vivo evaluation of combined CK2 inhibition and Irradiation in Human WiDr Tumours	TBB	WiDr (colon) xenograft, s.c.	BALB/c athymic nude mice, 6-8 weeks	i.p., 150 mg/kg, twice daily+ irradiation for 5 days	DMSO	marked and significantly delayed tumour growth	N/A	inhibition of CK2	[4]
Fused derivative	He 2022	NIR-II absorptive dithienopyrrole-thiadiazolobenzotriazole conjugated polymer for photoacoustic imaging-guided glioblastoma multiforme photothermal therapy	PT NPs, cRGD@PT NPs	GL261-luc cells (glioblastoma) injected in the right brain and C6 cells (glioma), orthotopic xenograft	female C57BL/6 and nude mice, 4–6 weeks	Control (PBS) + laser; (ii) cRGD@PT NPs (1 mg/Kg); (iii) cRGD@PT NPs (1 mg/Kg) + laser	PBS	cRGD@PT NPs with laser irradiation showed a prominent tumor reduction	similar body weight profiles with no meaningful changes	expression of CD31 and ki67 positive cells significantly decreased in cRGD@PT NPs + laser	[15]
N-substituted	Wu 2021	Design, synthesis, and biological evaluation of 3-(1-benzotriazole)-nor-β-lapachones as NQO1-directed antitumor agents	5k	HepG2 (hepatocellular) xenograft, s.c. in the right flank	female athymic nude mic, 7–8 weeks, 18–22g	i.v., 20 mg/kg, every two days for 19 consecutive days	saline	tumor size decreased by 2.1-fold; tumor weight	no toxicity observed	N/A	[11]

								decreased by 52.3% in comparison with control			
Organometallic complex	Li 2013	A new synthetic Cu(II) compound, $[Cu_3(p\text{-}3\text{-}bmb)_2Cl_4\cdot(CH_3OH)_2]_n$, inhibits tumor growth in vivo and in vitro	Cu (II)	sarcoma murine cancer (S180), i.p.	male mice, 18–22g	i.p.; 1, 5, 10 mg/kg/day with Cu(II) to 7 day, 1 mg/kg/day with Cisplatin to 7 day	cisplatin, saline	tumor growt inhibition: 1 mg/kg (42.29%), 5mg/kg (50.90%) and 10 mg/kg (59.86%)	no body weight changes, 10 mg/kg, Cu(II) compound migh induce some degree of hepatotoxicity	apoptosis, G1 phase arrest via cyclinD1/cdk4 pathway	[36]

Table S3. QUIN tool-Risk of bias (Reviewer 1).

First author's name and year of publication	Clearly stated aims/objectives	Detailed explanation of sample size calculation	Detailed explanation of sampling technique	Details of comparison group	Detailed explanation of methodology	Operator details	Randomization	Method of measurement of outcome	Outcome assessor details	Blinding	Statistical analysis	Presentation of results
Alraqa 2020	2	1	2	2	2	0	0	2	1	0	1	2
Anusha 2022	2	1	2	2	2	0	0	2	1	0	1	2
Aziz 2024	2	1	1	2	2	0	0	2	1	0	2	2
Borowiecki 2018	2	1	2	2	2	0	0	2	1	0	2	2
Chojnacka 2016	1	1	2	2	2	0	0	2	1	0	0	2
Chojnacki 2017	2	1	2	2	2	0	0	2	1	0	2	2

El-Asmy 2013	2	1	1	2	2	0	0	2	1	0	1	2
El-Kardocy 2020	2	0	2	2	2	0	0	2	1	0	1	2
Entezari 2013	1	1	2	2	2	0	0	2	1	0	2	2
Fabitha 2024	2	1	1	2	2	0	0	2	1	0	2	2
Fan 2020	2	1	2	2	2	0	0	2	1	0	2	2
Garton 2021	2	0	1	1	2	0	0	2	1	0	2	2
He 2022	2	1	2	2	2	0	0	2	1	0	2	2
Hu 2017	2	1	2	2	1	0	0	2	1	0	0	2
Ibba 2021	2	1	2	2	2	0	0	2	1	0	1	2
Kassab 2018	2	1	1	2	2	0	0	2	1	0	1	2
Khayyat 2021	2	1	2	2	2	0	0	2	1	0	1	2
Khodair 2019	2	1	2	2	2	0	0	2	1	0	2	2
Korcz 2018	2	1	2	1	2	0	0	2	1	0	1	2
Kumar 2020	1	1	2	2	2	0	0	2	1	0	2	2
Kuran 2020	2	1	1	2	2	0	0	2	1	0	2	2
Li 2013	2	1	2	2	2	0	0	2	1	0	1	2
Li 2019	2	0	2	2	2	0	0	2	1	0	1	2
Mansour 2014	2	1	2	2	2	0	0	2	1	0	1	2
Matheswari 2024	2	1	1	2	2	0	0	2	1	0	1	2
Mermer 2022	2	1	2	2	2	0	0	2	1	0	1	2
Mioc 2022	2	1	2	1	2	0	0	2	1	0	2	2
Onar 2019	2	1	1	2	2	0	0	2	1	0	0	2
Pogaku 2019	2	0	1	2	2	0	0	2	1	0	1	2
Qadri 2023	1	1	2	1	2	0	0	2	1	0	0	2
Shehata 2010	2	1	1	2	2	0	0	2	1	0	1	2
Stamou 2024	2	0	2	1	2	0	0	2	1	0	2	2
Swider 2015	2	0	2	2	2	0	0	2	1	0	1	2
Trembley 2019	1	1	2	2	2	0	0	2	1	0	2	2

Wang 2022	2	1	1	2	2	0	0	2	1	0	2	2
Wu 2021	2	0	2	2	2	0	0	2	1	0	0	2
Zhang 2013	2	0	2	2	2	0	0	2	1	0	0	2
Zhao 2015	2	1	1	2	2	0	0	2	1	0	0	2
Zhao 2017	2	0	1	2	2	0	0	2	1	0	0	2
Zoroddu 2024	2	0	2	1	2	0	0	2	1	0	2	2
Zwicker 2021	2	0	2	1	2	0	0	2	1	0	1	2

Table S4. QUIN tool-Risk of bias (Reviewer 2).

First author's name and year of publication	Clearly stated aims/objectives	Detailed explanation of sample size calculation	Detailed explanation of sampling technique	Details of comparison group	Detailed explanation of methodology	Operator details	Randomization	Method of measurement of outcome	Outcome assessor details	Blinding	Statistical analysis	Presentation of results
Alraqa 2020	1	1	2	2	2	0	0	2	1	0	1	2
Anusha 2022	2	1	2	2	2	0	0	2	1	0	1	2
Aziz 2024	2	1	2	2	2	0	0	2	1	0	2	2
Borowiecki 2018	2	1	2	2	2	0	0	2	1	0	2	2
Chojnacka 2016	2	1	2	2	2	0	0	2	1	0	0	2
Chojnacki 2017	2	1	2	2	2	0	0	2	1	0	2	2
El-Asmy 2013	2	1	1	2	2	0	0	2	1	0	1	2
El-Kardocy 2020	2	0	2	2	2	0	0	2	1	0	1	2
Entezari 2013	2	1	2	2	2	0	0	2	1	0	2	2
Fabitha 2024	2	1	2	2	2	0	0	2	1	0	2	2
Fan 2020	2	1	2	2	2	0	0	2	1	0	2	2
Garton 2021	2	0	1	1	2	0	0	2	1	0	2	2
He 2022	1	1	2	2	2	0	0	2	1	0	2	2
Hu 2017	2	1	2	2	2	0	0	2	1	0	0	2

Ibba 2021	2	1	2	2	2	0	0	2	1	0	1	2
Kassab 2018	2	1	2	2	2	0	0	2	1	0	1	2
Khayyat 2021	1	1	2	2	2	0	0	2	1	0	1	2
Khodair 2019	2	1	1	2	2	0	0	2	1	0	2	2
Korcz 2018	2	1	2	1	2	0	0	2	1	0	1	2
Kumar 2020	2	1	2	2	2	0	0	2	1	0	2	2
Kuran 2020	2	1	1	2	2	0	0	2	1	0	2	2
Li 2013	2	1	2	2	2	0	0	2	1	0	1	2
Li 2019	2	0	2	2	2	0	0	2	1	0	1	2
Mansour 2014	1	1	2	2	2	0	0	2	1	0	1	2
Matheswari 2024	2	1	2	2	2	0	0	2	1	0	1	2
Mermer 2022	2	1	2	2	2	0	0	2	1	0	1	2
Mioc 2022	2	1	2	1	2	0	0	2	1	0	2	2
Onar 2019	2	1	1	2	2	0	0	2	1	0	0	2
Pogaku 2019	2	0	1	2	2	0	0	2	1	0	1	2
Qadri 2023	2	1	2	2	2	0	0	2	1	0	0	2
Shehata 2010	2	1	1	2	2	0	0	2	1	0	1	2
Stamou 2024	2	0	2	1	2	0	0	2	1	0	2	2
Swider 2015	2	0	2	2	1	0	0	2	1	0	1	2
Trembley 2019	2	1	2	2	2	0	0	2	1	0	2	2
Wang 2022	2	1	1	2	2	0	0	2	1	0	2	2
Wu 2021	2	0	2	2	2	0	0	2	1	0	0	2
Zhang 2013	2	0	2	2	2	0	0	2	1	0	0	2
Zhao 2015	2	1	2	2	2	0	0	2	1	0	0	2
Zhao 2017	2	0	2	2	2	0	0	2	1	0	0	2
Zoroddu 2024	2	0	2	1	2	0	0	2	1	0	2	2
Zwicker 2021	2	0	2	1	2	0	0	2	1	0	1	2

Table S5. QUIN tool-Risk of bias (Team discussions).

First author's name and year of publication	Clearly stated aims/objectives	Detailed explanation of sample size calculation	Detailed explanation of sampling technique	Details of comparison group	Detailed explanation of methodology	Operator details	Randomization	Method of measurement of outcome	Outcome assessor details	Blinding	Statistical analysis	Presentation of results	Total score	Final score (%)	Risk of bias
Alraqa 2020	2	1	2	2	2	0	0	2	1	0	1	2	15	62.50	Medium
Anusha 2022	2	1	2	2	2	0	0	2	1	0	1	2	15	62.50	Medium
Aziz 2024	2	1	2	2	2	0	0	2	1	0	2	2	16	66.67	Medium
Borowiecki 2018	2	1	2	2	2	0	0	2	1	0	2	2	16	66.67	Medium
Chojnacka 2016	2	1	2	2	2	0	0	2	1	0	0	2	14	58.33	Medium
Chojnacki 2017	2	1	2	2	2	0	0	2	1	0	2	2	16	66.67	Medium
El-Asmy 2013	2	1	2	2	2	0	0	2	1	0	1	2	15	62.50	Medium
El-Kardocy 2020	2	0	2	2	2	0	0	2	1	0	1	2	14	58.33	Medium
Entezari 2013	2	1	2	2	2	0	0	2	1	0	2	2	16	66.67	Medium
Fabitha 2024	2	1	2	2	2	0	0	2	1	0	2	2	16	66.67	Medium
Fan 2020	2	1	2	2	2	0	0	2	1	0	2	2	16	66.67	Medium

Garton 2021	2	0	2	1	2	0	0	2	1	0	2	2	14	58.33	**Medium**
He 2022	2	1	2	2	2	0	0	2	1	0	2	2	16	66.67	**Medium**
Hu 2017	2	1	2	2	2	0	0	2	1	0	0	2	14	58.33	**Medium**
Ibba 2021	2	1	2	2	2	0	0	2	1	0	1	2	15	62.50	**Medium**
Kassab 2018	2	1	2	2	2	0	0	2	1	0	1	2	15	62.50	**Medium**
Khayyat 2021	2	1	2	2	2	0	0	2	1	0	1	2	15	62.50	**Medium**
Khodair 2019	2	1	2	2	2	0	0	2	1	0	2	2	16	66.67	**Medium**
Korcz 2018	2	1	2	1	2	0	0	2	1	0	1	2	14	58.33	**Medium**
Kumar 2020	2	1	2	2	2	0	0	2	1	0	2	2	16	66.67	**Medium**
Kuran 2020	2	1	2	2	2	0	0	2	1	0	2	2	16	66.67	**Medium**
Li 2013	2	1	2	2	2	0	0	2	1	0	1	2	15	62.50	**Medium**
Li 2019	2	0	2	2	2	0	0	2	1	0	1	2	14	58.33	**Medium**
Mansour 2014	2	1	2	2	2	0	0	2	1	0	1	2	15	62.50	**Medium**
Matheswari 2024	2	1	2	2	2	0	0	2	1	0	1	2	15	62.50	**Medium**
Mermer 2022	2	1	2	2	2	0	0	2	1	0	1	2	15	62.50	**Medium**
Mioc 2022	2	1	2	1	2	0	0	2	1	0	2	2	15	62.50	**Medium**

Onar 2019	2	1	2	2	2	0	0	2	1	0	0	2	14	58.33	**Medium**
Pogaku 2019	2	0	1	2	2	0	0	2	1	0	1	2	13	54.17	**Medium**
Qadri 2023	2	1	2	2	2	0	0	2	1	0	0	2	14	58.33	**Medium**
Shehata 2010	2	1	2	2	2	0	0	2	1	0	1	2	15	62.50	**Medium**
Stamou 2024	2	0	2	1	2	0	0	2	1	0	2	2	14	58.33	**Medium**
Swider 2015	2	0	2	2	2	0	0	2	1	0	1	2	14	58.33	**Medium**
Trembley 2019	2	1	2	2	2	0	0	2	1	0	2	2	16	66.67	**Medium**
Wang 2022	2	1	2	2	2	0	0	2	1	0	2	2	16	66.67	**Medium**
Wu 2021	2	0	2	2	2	0	0	2	1	0	0	2	13	54.17	**Medium**
Zhang 2013	2	0	2	2	2	0	0	2	1	0	0	2	13	54.17	**Medium**
Zhao 2015	2	1	2	2	2	0	0	2	1	0	0	2	14	58.33	**Medium**
Zhao 2017	2	0	2	2	2	0	0	2	1	0	0	2	13	54.17	**Medium**
Zoroddu 2024	2	0	2	1	2	0	0	2	1	0	2	2	14	58.33	**Medium**
Zwicker 2021	2	0	2	1	2	0	0	2	1	0	1	2	13	54.17	**Medium**

Table S6. SYRCLE Risk of bias (Reviewer 1).

	Selection bias			Performance bias		Detection bias		Attrition bias	Reporting bias	Other
**First author's name and year of publication**	**Sequence generation**	**Baseline characteristics**	**Allocation concealment**	**Random housing**	**Blinding**	**Random outcome assessment**	**Blinding**	**Incomplete outcome data**	**Selective outcome reporting**	**Report of ethical approval for the animal study**
Fan 2020	Unclear	Low	High	Low	High	Low	High	Low	Low	Low
Trembley 2019	Unclear	Unclear	High	Unclear	High	High	High	Low	Low	High
Zwicker 2021	Unclear	Unclear	High	Unclear	High	Low	High	Low	Low	Low
He 2022	Unclear	Low	High	Unclear	High	Low	High	Low	Low	Low
Wu 2021	Unclear	Low	High	Unclear	High	Low	High	Low	Low	Low
Li 2013	Unclear	Unclear	High	Unclear	High	Low	High	Low	Low	Low

Table S7. SYRCLE Risk of bias (Reviewer 2).

	**Selection bias**			**Performance bias**		**Detection bias**		**Attrition bias**	**Reporting bias**	**Other**
**First author's name and year of publication**	**Sequence generation**	**Baseline characteristics**	**Allocation concealment**	**Random housing**	**Blinding**	**Random outcome assessment**	**Blinding**	**Incomplete outcome data**	**Selective outcome reporting**	**Report of ethical approval for the animal study**
Fan 2020	Unclear	Low	High	Low	High	Low	High	Low	Low	Low
Trembley 2019	Unclear	Unclear	High	Unclear	High	High	High	Low	Low	High
Zwicker 2021	Unclear	Unclear	High	Unclear	High	Low	High	Low	Low	Low
He 2022	Unclear	Low	High	Unclear	High	Low	High	Low	Low	Low
Wu 2021	Unclear	Low	High	Unclear	High	Low	High	Low	Low	Low
Li 2013	Unclear	Unclear	High	Unclear	High	Low	High	Low	Low	Low

1. Fan, Y.; Huang, Z.; Wang, X.; Ma, Y.; Li, Y.; Yang, S.; Shi, Y. Discovery of 12O—A Novel Oral Multi-Kinase Inhibitor for the Treatment of Solid Tumor. *Molecules* **2020**, *25*, 5199. https://doi.org/10.3390/molecules25215199.
2. Shehata, M Shehata, M.; Schnabl, S.; Demirtas, D.; Hilgarth, M.; Hubmann, R.; Ponath, E.; Badrnya, S.; Lehner, C.; Hoelbl, A.; Duechler, M.; et al. Reconstitution of PTEN Activity by CK2 Inhibitors and Interference with the PI3-K/Akt Cascade Counteract the Antiapoptotic Effect of Human Stromal Cells in Chronic Lymphocytic Leukemia. *Blood* **2010**, *116*, 2513–2521. https://doi.org/10.1182/blood-2009-10-248054.
3. Trembley, J.H.; Kren, B.T.; Abedin, M.J.; Shaughnessy, D.P.; Li, Y.; Dehm, S.M.; Ahmed, K. CK2 Pro-Survival Role in Prostate Cancer Is Mediated via Maintenance and Promotion of Androgen Receptor and NFκB P65 Expression. *Pharmaceuticals* **2019**, *12*, 89. https://doi.org/10.3390/ph12020089.
4. Zwicker, F.; Hauswald, H.; Weber, K.J.; Debus, J.; Huber, P.E. In Vivo Evaluation of Combined CK2 Inhibition and Irradiation in Human WiDr Tumours. *In Vivo* **2021**, *35*, 111–117. https://doi.org/10.21873/INVIVO.12238.
5. Chojnacki, K.; Wińska, P.; Skierka, K.; Wielechowska, M.; Bretner, M. Synthesis, in Vitro Antiproliferative Activity and Kinase Profile of New Benzimidazole and Benzotriazole Derivatives. *Bioorg. Chem.* **2017**, *72*, 1–10. https://doi.org/10.1016/j.bioorg.2017.02.017.
6. El-Kardocy, A.; Mostafa, Y.A.; Mohamed, N.G.; Abo-Zeid, M.N.; Hassan, N.A.; Hetta, H.F.; Abdel-Aal, A.B.M. CK2 Inhibition, Lipophilicity and Anticancer Activity of New: N 1versus N 2-Substituted Tetrabromobenzotriazole Regioisomers. *New J. Chem.* **2020**, *44*, 13007–13017. https://doi.org/10.1039/d0nj01194k.
7. Łukowska-Chojnacka, E.; Wińska, P.; Wielechowska, M.; Bretner, M. Synthesis of Polybrominated Benzimidazole and Benzotriazole Derivatives Containing a Tetrazole Ring and Their Cytotoxic Activity. *Monatshefte Fur Chem.* **2016**, *147*, 1789–1796. https://doi.org/10.1007/s00706-016-1785-8.
8. Entezari, M.; Safari, M.; Hekmati, M.; Hekmat, S.; Azin, A. Modification of Carboxylated Multiwall Nanotubes with Benzotriazole Derivatives and Study of Their Anticancer Activities. *Med. Chem. Res.* **2014**, *23*, 487–495. https://doi.org/10.1007/s00044-013-0668-3.
9. Swider, R.; Masłyk, M.; Zapico, J.M.; Coderch, C.; Panchuk, R.; Skorokhyd, N.; Schnitzler, A.; Niefind, K.; De Pascual-Teresa, B.; Ramos, A. Synthesis, Biological Activity and Structural Study of New Benzotriazole-Based Protein Kinase CK2 Inhibitors. *RSC Adv.* **2015**, *5*, 72482–72494. https://doi.org/10.1039/c5ra12114k.
10. Ibba, R.; Piras, S.; Corona, P.; Riu, F.; Loddo, R.; Delogu, I.; Collu, G.; Sanna, G.; Caria, P.; Dettori, T.; et al. Synthesis, Antitumor and Antiviral In Vitro Activities of New Benzotriazole-Dicarboxamide Derivatives. *Front. Chem.* **2021**, *9*, 660424. https://doi.org/10.3389/fchem.2021.660424.
11. Wu, L.Q.; Ma, X.; Liu, Z.P. Design, Synthesis, and Biological Evaluation of 3-(1-Benzotriazole)-nor-β-Lapachones as NQO1-Directed Antitumor Agents. *Bioorg. Chem.* **2021**, *113*, 104995. https://doi.org/10.1016/j.bioorg.2021.104995.
12. Borowiecki, P.; Wińska, P.; Bretner, M.; Gizińska, M.; Koronkiewicz, M.; Staniszewska, M. Synthesis of Novel Proxyphylline Derivatives with Dual Anti-Candida Albicans and Anticancer Activity. *Eur. J. Med. Chem.* **2018**, *150*, 307–333. https://doi.org/10.1016/j.ejmech.2018.02.077.
13. Li, Q.; Liu, G.; Wang, N.; Yin, H.; Li, Z. Synthesis and Anticancer Activity of Benzotriazole Derivatives. *J. Heterocycl. Chem.* **2020**, *57*, 1220–1227. https://doi.org/10.1002/jhet.3859.
14. Wang, X.; Zhang, M.; Xiong, X.Q.; Yang, H.; Wang, P.; Zhang, K.; Awadasseid, A.; Narva, S.; Wu, Y.L.; Zhang, W. Design, Synthesis and Bioactivity of Novel Naphthalimide-Benzotriazole Conjugates against A549 Cells via Targeting BCL2 G-Quadruplex and Inducing Autophagy. *Life Sci.* **2022**, *302*, 120651. https://doi.org/10.1016/j.lfs.2022.120651.
15. He, Y.; Pan, Y.; Zhao, X.; Fan, W.; Cai, Y.; Mou, X. NIR-II Absorptive Dithienopyrrole-Thiadiazolobenzotriazole Conjugated Polymer for Photoacoustic Imaging-Guided Glioblastoma Multiforme Photothermal Therapy. *Acta Biomater.* **2022**, *152*, 546–561. https://doi.org/10.1016/j.actbio.2022.07.045.

16. Alraqa, S.Y.; Alharbi, K.; Aljuhani, A.; Rezki, N.; Aouad, M.R.; Ali, I. Design, Click Conventional and Microwave Syntheses, DNA Binding, Docking and Anticancer Studies of Benzotriazole-1,2,3-Triazole Molecular Hybrids with Different Pharmacophores. *J. Mol. Struct.* **2021**, *1225*, 129192. https://doi.org/10.1016/j.molstruc.2020.129192.
17. Anusha, D.; Susithra, E. Synthesis and Biological Evaluation of Substituted Mannich Bases of Benzotriazole Derivatives As Anticancer Agents. *Eur. Chem. Bull.* **2022**, *11*, 62–71. https://doi.org/10.31838/ecb/2022.11.10.009.
18. Aziz, M.; Ejaz, S.A.; Channar, P.A.; Alkhathami, A.G.; Qadri, T.; Hussain, Z.; Hussaain, M.; Ujan, R. Identification of Dimethyl 2,2′-((Methylenebis(2-(2H-Benzo[d][1,2,3]Triazol-2-Yl)-4-(2,4,4-Trimethylpentan-2-Yl)-6,1phenylene))Bis(Oxy))Diacetate (TAJ4) as Antagonist of NEK-Family: A Future for Potential Drug Discovery. *BMC Cancer* **2024**, *24*, 1521. https://doi.org/10.1186/s12885-024-13269-4.
19. Fabitha, K.; Kallingal, A.; Maciejewska, N.; Arya, C.G.; Chandrakanth, M.; Thomas, N.M.; Li, Y.; Gondru, R.; Munikumar, M.; Banothu, J. Novel Fused Pyran Derivatives Induce Apoptosis and Target Cell Cycle Progression in Anticancer Efficacy against Multiple Cell Lines. *New J. Chem.* **2024**, *48*, 8038–8054. https://doi.org/10.1039/D4NJ00824C.
20. Garton, C.S.; Derose, N.K.; Dominguez, D.; Turbi-Henderson, M.L.; Lehr, A.L.; Padilla, A.D.; Twining, S.D.; Casas, S.; Alozie, C.O.; Gucwa, A.L.; et al. Synthesis and Antiproliferative Evaluation of 2-Deoxy-n-Glycosylbenzotriazoles/Imidazoles. *Molecules* **2021**, *26*, 3742. https://doi.org/10.3390/molecules26123742.
21. Kassab, A.E.; Hassan, R.A. Novel Benzotriazole N-Acylarylhydrazone Hybrids: Design, Synthesis, Anticancer Activity, Effects on Cell Cycle Profile, Caspase-3 Mediated Apoptosis and FAK Inhibition. *Bioorg. Chem.* **2018**, *80*, 531–544. https://doi.org/10.1016/j.bioorg.2018.07.008.
22. Khayyat, A.N.; Mohamed, K.O.; Malebari, A.M.; El-malah, A. Substituted Imidazole-Thione Linked. *Molecules* **2021**, *26*, 5983.
23. Khodair, A.I.; Attia, A.M.; Gendy, E.A.; Elshaier, Y.A.M.M.; El-Magd, M.A. Discovery of New S -Glycosides and N -Glycosides of Pyridine-biphenyl System with Antiviral Activity and Induction of Apoptosis in MCF 7 Cells. *J. Heterocycl. Chem.* **2019**, *56*, 1733–1747. https://doi.org/10.1002/jhet.3527.
24. Khodair, A.I.; Attia, A.M.; Gendy, E.A.; Elshaier, Y.A.M.M.; El-Magd, M.A. Discovery of New S -Glycosides and N -Glycosides of Pyridine-biphenyl System with Antiviral Activity and Induction of Apoptosis in MCF 7 Cells. *J. Heterocycl. Chem.* **2019**, *56*, 1733–1747. https://doi.org/10.1002/jhet.3527.
25. Rajesh Kumar, M.; Violet Dhayabaran, V.; Sudhapriya, N.; Manikandan, A.; Gideon, D.A.; Annapoorani, S. P-TSA.H2O Mediated One-Pot, Multi-Component Synthesis of Isatin Derived Imidazoles as Dual-Purpose Drugs against Inflammation and Cancer. *Bioorg. Chem.* **2020**, *102*, 104046. https://doi.org/10.1016/j.bioorg.2020.104046.
26. Kuran, D.; Flis, S.; Antoszczak, M.; Piskorek, M.; Huczyński, A. Ester Derivatives of Salinomycin Efficiently Eliminate Breast Cancer Cells via ER-Stress-Induced Apoptosis. *Eur. J. Pharmacol.* **2021**, *893*, 173824. https://doi.org/10.1016/j.ejphar.2020.173824.
27. Pon Matheswari, P.; Ilavarasi Jeyamalar, J.; Iruthayaraj, A.; Ravindran Durai Nayagam, B. Synthesis, Structural, Multitargeted Molecular Docking Analysis of Anti-Cancer, Anti-Tubercular, DNA Interactions of Benzotriazole Based Macrocyclic Ligand. *Bioorg. Chem.* **2024**, *147*, 107361. https://doi.org/10.1016/j.bioorg.2024.107361.
28. Mermer, A.; Volkan Bulbul, M.; Mervenur Kalender, S.; Keskin, I.; Tuzun, B.; Emre Eyupoglu, O. Benzotriazole-Oxadiazole Hybrid Compounds: Synthesis, Anticancer Activity, Molecular Docking and ADME Profiling Studies. *J. Mol. Liq.* **2022**, *359*, 119264. https://doi.org/10.1016/j.molliq.2022.119264.
29. Mioc, M.; Mioc, A.; Prodea, A.; Milan, A.; Balan-Porcarasu, M.; Racoviceanu, R.; Ghiulai, R.; Iovanescu, G.; Macasoi, I.; Draghici, G.; et al. Novel Triterpenic Acid—Benzotriazole Esters Act as Pro-Apoptotic Antimelanoma Agents. *Int. J. Mol. Sci.* **2022**, *23*, 9992. https://doi.org/10.3390/ijms23179992.

30. Pogaku, V.; Krishna, V.S.; Balachandran, C.; Rangan, K.; Sriram, D.; Aoki, S.; Basavoju, S. The Design and Green Synthesis of Novel Benzotriazoloquinolinyl Spirooxindolopyrrolizidines: Antimycobacterial and Antiproliferative Studies. *New J. Chem.* **2019**, *43*, 17511–17520. https://doi.org/10.1039/C9NJ03802G.
31. Qadri, T.; Aziz, M.; Channar, P.A.; Ejaz, S.A.; Hussain, M.; Attaullah, H.M.; Ujan, R.; Hussain, Z.; Zehra, T.; Saeed, A.; et al. Synthesis, Biological Evaluation and in Silico Investigations of Benzotriazole Derivatives as Potential Inhibitors of NIMA Related Kinase. *RSC Adv.* **2023**, *13*, 33826–33843. https://doi.org/10.1039/D3RA06149C.
32. Zhang, S.; Luo, Y.; He, L.-Q.; Liu, Z.-J.; Jiang, A.-Q.; Yang, Y.-H.; Zhu, H.-L. Synthesis, Biological Evaluation, and Molecular Docking Studies of Novel 1,3,4-Oxadiazole Derivatives Possessing Benzotriazole Moiety as FAK Inhibitors with Anticancer Activity. *Bioorg. Med. Chem.* **2013**, *21*, 3723–3729. https://doi.org/10.1016/j.bmc.2013.04.043.
33. Zoroddu, S.; Sanna, L.; Bordoni, V.; Weidong, L.; Gadau, S.D.; Carta, A.; Kelvin, D.J.; Bagella, L. Identification of 3-Aryl-1-Benzotriazole-1-Yl-Acrylonitrile as a Microtubule-Targeting Agent (MTA) in Solid Tumors. *Int. J. Mol. Sci.* **2024**, *25*, 5704. https://doi.org/10.3390/ijms25115704.
34. El-Asmy, H.A.; Butler, I.S.; Mouhri, Z.S.; Jean-Claude, B.J.; Emmam, M.S.; Mostafa, S.I. Zinc(II), Ruthenium(II), Rhodium(III), Palladium(II), Silver(I), Platinum(II) and MoO 2 2 + Complexes of 2-(2′-Hydroxy-5′- Methylphenyl)-Benzotriazole as Simple or Primary Ligand and 2,2′- Bipyridyl, 9,10-Phenanthroline or Triphenylphosphine as Secondary . *J. Mol. Struct.* **2014**, *1059*, 193–201. https://doi.org/10.1016/j.molstruc.2013.11.039.
35. Hu, J.; Guo, Y.; Zhao, J.; Zhang, J. In Vitro Antitumor Activity of Novel Benzimidazole-Based Cu(II) Complexes. *Bioorganic Med. Chem.* **2017**, *25*, 5733–5742. https://doi.org/10.1016/j.bmc.2017.08.053.
36. Li, R.; Cui, B.; Li, Y.; Zhao, C.; Jia, N.; Wang, C.; Wu, Y.; Wen, A. A New Synthetic Cu(II) Compound, [Cu3(p-3-Bmb)2Cl4·(CH3OH)2] , Inhibits Tumor Growth in Vivo and in Vitro. *Eur. J. Pharmacol.* **2014**, *724*, 77–85. https://doi.org/10.1016/j.ejphar.2013.12.007.
37. Mansour, A.M.; Mohamed, M.F. Complexes of N-(2-Thiazolyl)-1H-Benzotriazole-1-Carbothioamide with Pd(II), Pt(II), and Zn(II): Spectral, DFT, Cytotoxicity and Anti-Angiogenic Effect on MCF-7 Cell Line. *Inorganica Chim. Acta* **2014**, *423*, 373–383. https://doi.org/10.1016/j.ica.2014.08.034.
38. Onar, G.; Gürses, C.; Karataş, M.O.; Balcıoğlu, S.; Akbay, N.; Özdemir, N.; Ateş, B.; Alıcı, B. Palladium(II) and Ruthenium(II) Complexes of Benzotriazole Functionalized N-Heterocyclic Carbenes: Cytotoxicity, Antimicrobial, and DNA Interaction Studies. *J. Organomet. Chem.* **2019**, *886*, 48–56. https://doi.org/10.1016/j.jorganchem.2019.02.013.
39. Stamou, C.; Gourdoupi, C.; Dechambenoit, P.; Papaioannou, D.; Piperigkou, Z.; Lada, Z.G. Antiproliferative Activity of an Organometallic Sn(IV) Coordination Compound Based on 1-Methylbenzotriazole against Human Cancer Cell Lines. *Chemistry* **2024**, *6*, 1189–1200. https://doi.org/10.3390/chemistry6050068.
40. Zhao, J.; Guo, Y.; Hu, J.; Yu, H.; Zhi, S.; Zhang, J. Potential Anticancer Activity of Benzimidazole-Based Mono/Dinuclear Zn(II) Complexes towards Human Carcinoma Cells. *Polyhedron* **2015**, *102*, 163–172. https://doi.org/10.1016/j.poly.2015.09.057.
41. Zhao, J.A.; Yu, H.B.; Zhi, S.C.; Mao, R.N.; Hu, J.Y.; Wang, X.X. Synthesis, Chemical Nuclease Activity, and in Vitro Cytotoxicity of Benzimidazole-Based Cu(II)/Co(II) Complexes. *Chin. Chem. Lett.* **2017**, *28*, 1539–1546. https://doi.org/10.1016/j.cclet.2017.03.025.
